# Optimizing Biologic Treatment Selection in Chronic Rhinosinusitis with Nasal Polyps: A Network Meta-Analysis of Efficacy and Safety Across 22 RCTs

**DOI:** 10.3390/ph18101455

**Published:** 2025-09-28

**Authors:** Alaa Safia, Ashraf Khater, Uday Abd Elhadi, Shlomo Merchavy, Marwan Karam

**Affiliations:** Department of Otolaryngology, Rhinology Unit, Ziv Medical Center, Safed 1311001, Israel

**Keywords:** biological therapies, chronic rhinosinusitis, nasal polyps, network meta-analysis

## Abstract

**Background/Objectives:** Biological therapies have emerged as targeted treatments for chronic rhinosinusitis with nasal polyps (CRSwNP), yet direct comparisons between agents remain limited. This network meta-analysis (NMA) aimed to evaluate and rank the efficacy and safety of biological therapies for CRSwNP in adult patients. **Methods:** We conducted a systematic review and NMA of randomized controlled trials (RCTs) assessing biological therapies for CRSwNP. A literature search was conducted through July 2025. Eligible RCTs compared approved or investigational biologics with a placebo and reported clinical, functional, or safety outcomes in adults with CRSwNP. The mean differences (MDs) in the least-squares mean change from baseline were used for continuous outcomes, and odds ratios (ORs) were used for binary outcomes. A frequentist random-effects model was used to estimate pooled effects and treatment rankings. SUCRA values and rank probabilities were derived to determine the relative efficacy and safety. **Results:** A total of 22 RCTs (46 reports; 4068 patients) evaluating eight biologics were included. Dupilumab consistently ranked among the top three agents across most efficacy outcomes, including nasal polyp score (NPS), nasal congestion score (NCS), SNOT-22, UPSIT, and endoscopic scores. CM310 and Tezepelumab also demonstrated strong performance in objective and symptom-based outcomes. For responder outcomes, CM310 was ranked best in minimal clinically important differences across multiple domains. PF-06817024 ranked best in minimizing any adverse events and serious adverse events. The placebo ranked worst across nearly all endpoints. **Conclusions:** Dupilumab, CM31 0, and Tezepelumab exhibit the most favorable efficacy profiles across multiple CRSwNP domains, while all drugs show a nearly similar safety profile.

## 1. Introduction

Chronic rhinosinusitis with nasal polyps (CRSwNP) is a persistent inflammatory disease of the nasal and paranasal sinuses, characterized by bilateral polypoid growth that obstructs airflow and impairs mucociliary function [1]. Patients often experience substantial symptom burden, including nasal obstruction, facial pressure, hyposmia or anosmia, and decreased quality of life [2]. CRSwNP frequently coexists with asthma and allergic rhinitis, and is commonly associated with type 2 (Th2) inflammation [3].

The underlying immunopathogenesis involves the upregulation of interleukin (IL)-4, IL-5, and IL-13, leading to eosinophilic infiltration and persistent mucosal inflammation [4]. Traditional therapies—including intranasal corticosteroids (INCS), short courses of systemic corticosteroids (SCS), and endoscopic sinus surgery—are effective for many patients, yet relapse is common, and a subset remains refractory to conventional interventions [5].

Recent advances have introduced biological therapies that selectively target type 2 inflammatory pathways, offering a precision medicine approach to CRSwNP [6]. Multiple randomized controlled trials (RCTs) have demonstrated the efficacy of monoclonal antibodies such as dupilumab, omalizumab, and mepolizumab in improving polyp size, sinonasal symptoms, olfactory function, and health-related quality of life. However, head-to-head comparisons are lacking, and questions remain regarding the relative efficacy and safety of different biologics.

A prior network meta-analysis (NMA) by Oykhman et al. [7] provided a comparative synthesis of several biologics and aspirin desensitization (ASA-D) in CRSwNP, identifying dupilumab as one of the most effective therapies. However, that study did not include recently evaluated biologics such as CM310 and PF-06817024 and combined different therapeutic classes, potentially obscuring class-specific effects. Furthermore, it did not explore SNOT-22 subdomains or responder thresholds, nor did it apply ranking-based frameworks to aid clinical decision-making. Additionally, several trials have been published since then [8,9].

To address these limitations, we conducted an updated NMA of biologic therapies for CRSwNP. This analysis incorporates data from 22 RCTs, including two newly published trials, and applies a frequentist random-effects model to assess both continuous and binary outcomes. Our focus is limited to comparisons with the placebo, and treatment efficacy is evaluated using least-squares mean changes from baseline. Additionally, we provide SUCRA-based treatment rankings across a wide range of domains, including SNOT-22 subdomains, olfaction, imaging, and responder outcomes. This study aims to provide a more granular and up-to-date assessment of the comparative effectiveness and safety of biologics for CRSwNP.

## 2. Materials and Methods

### 2.1. Protocol Registration and Search Strategy

This study was a NMA of randomized controlled trials (RCTs) comparing the efficacy and safety of biological therapies for CRSwNP. We followed the PRISMA guidelines for systematic reviews and the PRISMA extension for NMAs [10]. The study protocol was registered with PROSPERO (CRD42024550348). A comprehensive literature search was conducted across PubMed, Web of Science, Scopus, Cochrane Library, and clinicaltrials.gov, as well as the first 200 records on Google Scholar, through May 2024. Additionally, manual searches of references and citation tracking were performed. Non-English trials were also included [11]. The detailed search criteria are provided in Table S1. An updated search was performed in July 2025, yielding two additional RCTs [8,9].

### 2.2. Inclusion and Exclusion Criteria

According to the PICOS framework [12], we included RCTs evaluating biological therapies (Dupilumab, Mepolizumab, Omalizumab, Reslizumab, Benralizumab, CM310, Tezepelumab, and PF-06817024) for CRSwNP. Trials were required to compare biological therapies with placebo or other biologics, and to report efficacy or safety outcomes in adult patients with CRSwNP, with or without comorbid asthma. Studies with non-randomized designs, abstract-only publications, conference presentations, and non-original research were excluded.

### 2.3. Study Selection and Data Extraction

Two independent reviewers screened titles and abstracts and assessed full-text articles for eligibility. Discrepancies were resolved by discussion or a third reviewer. Data were extracted using a standardized form, capturing study characteristics (author, year, country, design), patient characteristics (age, gender, CRSwNP duration), intervention details (biological agent, dose, duration), and outcomes.

Key outcomes spanned multiple domains, including symptom severity and quality of life [Nasal Polyp Score (NPS), Nasal Congestion Score (NCS), Lund–Mackay CT score, and the Total SNOT-22 score], responder analysis (Minimal Clinically Important Difference—MCID across used measures), and safety profile (all reported adverse events [AEs] and serious AEs (SAEs)).

### 2.4. Risk of Bias Assessment

Risk of bias was assessed using the Cochrane Risk of Bias tool (RoB 2, revised version, 2019) across multiple domains: random sequence generation, allocation concealment, blinding, incomplete data, selective reporting, and other biases.

### 2.5. Statistical Analysis

All statistical analyses were conducted using Stata (version 18, StataCorp LLC., College Station, TX, USA) and R software (version 4.0.3, R Foundation for Statistical Computing, Vienna, Austria). The NMA was performed using the network and mvmeta packages in Stata, adopting a frequentist framework with a multivariate random-effects model.

For continuous outcomes, the primary effect measure was the mean difference (MD) in least-square (LS) mean change from baseline, as reported in the trials [13]. This approach was selected to reflect treatment-specific change over time and ensure comparability across studies with different baseline scores and follow-up durations. For trials reporting multiple timepoints, the last observation carried forward (LOCF) method was applied to retain the longest available follow-up per outcome.

For binary outcomes, odds ratios (ORs) with 95% confidence intervals (CIs) were calculated. Network meta-analysis enabled both direct and indirect comparisons between all treatments using placebo as the reference group.

Treatment rankings were generated using the network rank command. For each outcome, treatments were ordered based on the observed rank position. SUCRA values were computed but only selectively reported to avoid misinterpretation when spread across multiple ranking levels.

Inconsistency between direct and indirect evidence was evaluated using the node-splitting approach. Statistical heterogeneity was assessed using the I^2^ statistic, with values greater than 50% considered substantial. Sensitivity analyses included the inspection of Galbraith plots to identify outliers, which were reviewed and excluded if they distorted the pooled effect estimates. Funnel plots, Egger’s test, and Begg’s test were used to assess the potential for publication bias in binary outcomes [14,15].

## 3. Results

### 3.1. Literature Review

**Figure 1:**
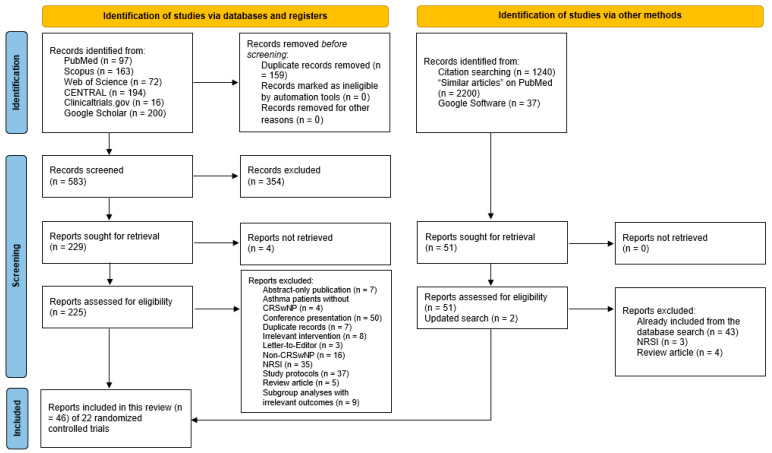
A PRISMA flow diagram showing the results of the literature search.

The literature search identified 742 citations (Figure 1). After removing 159 duplicates, 583 articles were screened, and 354 were excluded based on their title and abstract. Four articles could not be found, leaving 229 articles eligible for full-text screening. The reasons for exclusion are provided in Table S2. The updated search yielded two additional RCTs [8,9].

A total of 46 reports [8,9,11,16,17,18,19,20,21,22,23,24,25,26,27,28,29,30,31,32,33,34,35,36,37,38,39,40,41,42,43,44,45,46,47,48,49,50,51,52,53,54,55,56] from 22 RCTs met the inclusion criteria, with 1 trial being identified manually [57]. These trials investigated the efficacy of various biological therapies for CRSwNP, including Dupilumab, Reslizumab, Benralizumab, CM310, Tezepelumab, Omalizumab, and Mepolizumab, with a total of 4068 patients analyzed. A complete description of the diagnostic criteria and interventions used is provided in Table 1.

The baseline data on comorbid asthma, prior NP surgery, CRSwNP duration, and patient demographics are summarized in Table 2. A summary of all outcomes that were not eligible for meta-analysis are provided in Table S3. The number of patients analyzed in each outcome is presented in Table S4.

### 3.2. Risk of Bias Assessment

**Figure 2:**
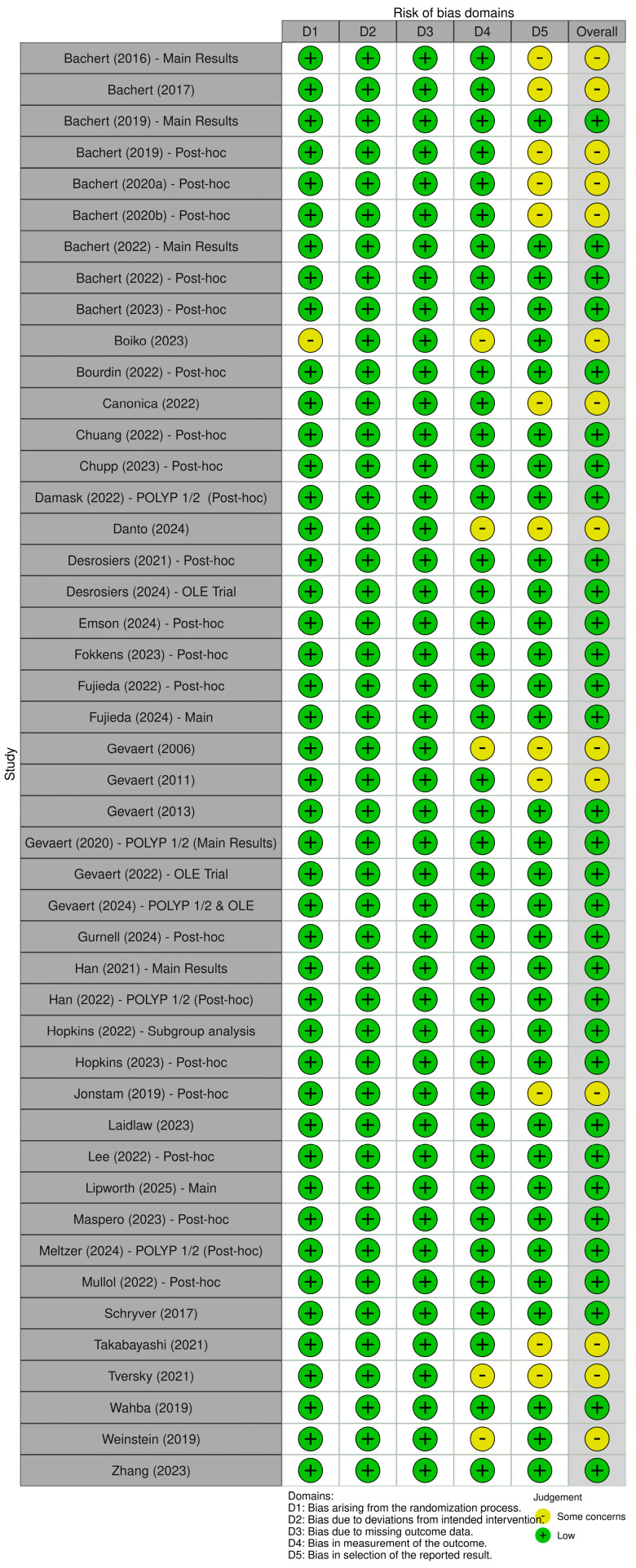
A summary graph of the risk of bias assessment of included randomized controlled trials [8,9,11,16,17,18,19,20,21,22,23,24,25,26,27,28,29,30,31,32,33,34,35,36,37,38,39,40,41,42,43,44,45,46,47,48,49,50,51,52,53,54,55,56,57].

Figure 2 shows the risk of bias assessment, where 33 studies had a low risk, and 13 had some concerns [8,9,11,16,17,18,20,21,22,23,25,48,49,50,51,58], mainly due to unregistered protocols and small sample sizes. The questionable methodology included five trials with small sample sizes (i.e., post hoc, subgroup analyses) leading to unreliable outcome measurements [11,16,17,20,23].

### 3.3. Characteristics of Included Trials

The RCTs included reports from various trials such as MERIT [8], WAYPOINT [9], OSTRO Trial [27,41], POLYP 1-2 and OLE “open-label extension” Trials [28,29,30,31,32,43], QUEST Trial [34,35,36], SINUS 24 and SINUS 52 Trials [25,26,31,37,38,39,40,42,43], SYNAPSE and OLE Trial [44,45,46,47,57], and a Phase IIa Trial (NCT01920893) [25,48,49,50,51]. The remaining data were pooled from small-scale RCTs. The follow-up duration varied from 3 weeks to 76 weeks, with a total of 2225 patients receiving biological therapies and 1855 patients receiving placebo or standard care. These reports were included either because they reported additional outcomes not reported in the original trials or reported the same outcomes but in different timepoints. All, except for one study [11], had placebo as the control group (Figure S1).

### 3.4. Efficacy Measures

#### 3.4.1. NPS

**Figure 3:**
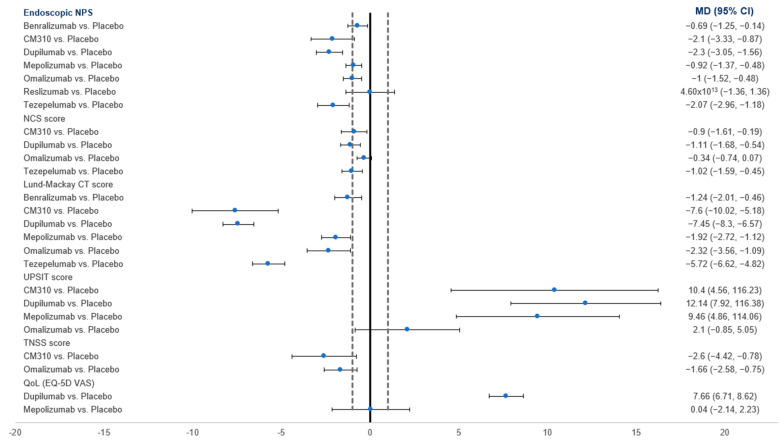
A forest plot showing the summary meta-analytic estimates (mean difference) from the placebo-focused comparisons with other biologic drugs regarding endoscopic NPS, NCS, Lund-Mackay CT score, UPSIT score, and TNSS score.

Compared to the placebo, all drugs showed a significantly greater reduction in NPS, except for reslizumab (Figure 3). Teszepelumab exhibited the greatest reduction (MD = −2.07; 95% CI: −2.96, −1.18).

In the network analysis, dupilumab was ranked the best treatment (SUCRA = 46.8%), followed by Tezepelumab (SUCRA = 35.9%) and Omalizumab (SUCRA = 45.6%), respectively, while reslizumab showed the worst outcome (SUCRA = 52.3%) (Table 3).

#### 3.4.2. NCS

Compared to the placebo, CM310, dupilumab (MD = −1.11; 95%CI: −1.68, −0.54), and Tezepelumab showed a significantly greater reduction in NCS, while Omalizumab showed no difference (Figure 3). In the network analysis, dupilumab (SUCRA = 45.7%) was ranked the best, followed by Tezepelumab (SUCRA = 36.7%), while the placebo was the worst (SUCRA = 94%) (Table 3).

#### 3.4.3. Lund–Mackay CT Score

Compared to the placebo, all drugs showed a significantly greater reduction in CT score, with dupilumab exhibiting the greatest reduction (MD = −7.45; 95%CI: −10.02, −5.18) (Figure 3). In the network analysis, CM310 (SUCRA = 54.5%) was ranked the best, followed by dupilumab (SUCRA = 54.1%) and Tezepelumab (SUCRA = 93%), respectively. The placebo was ranked the worst (SUCRA = 99.9%) (Table 3).

#### 3.4.4. SNOT-22 Score

**Figure 4:**
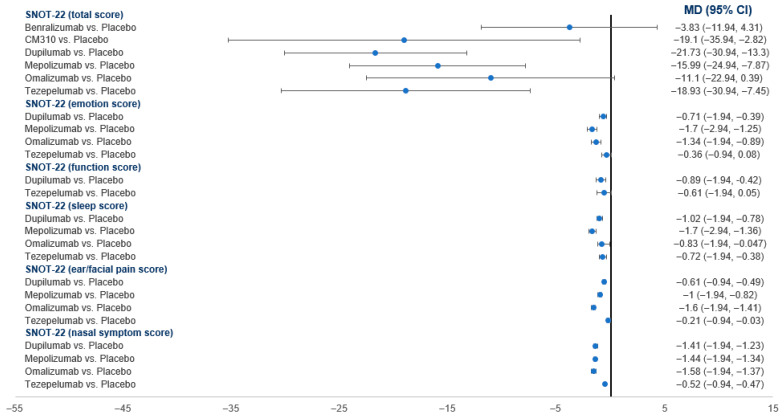
A forest plot showing the summary meta-analytic estimates (mean difference) from the placebo-focused comparisons with other biologic drugs regarding the total SNOT-22 score and its subdomains.

Regarding the total score, compared to the placebo, all drugs showed a significantly greater reduction except for Omalizumab, which showed no difference (Figure 4). Dupilumab showed the greatest reduction (MD = −21.73; 95% CI: −30.16, −13.3). In the network analysis, dupilumab (SUCRA = 38.1%) was ranked the best, followed by mepolizumab (SUCRA = 29.3%). Placebo was ranked the worst (SUCRA = 79.1%) (Table 3).

In terms of SNOT-22 subdomains (Figure 4), Tezepelumab did not differ from the placebo in the emotion and function scores. Other drugs (dupilumab, mepolizumab, omalizumab) showed a significantly greater reduction instead. In the network analysis, Omalizumab was ranked the best in the ear/facial pain (SUCRA = 100%) and nasal symptom subdomains (SUCRA = 81.6%), while Mepolizumab was ranked the best in the emotion (SUCRA = 87.1%) and sleep subdomains (SUCRA = 99.9%), and dupilumab was ranked the best in the function subdomain (SUCRA = 76.1%). In all subdomains, placebo was ranked the worst (Table 3).

#### 3.4.5. VAS Score

**Figure 5:**
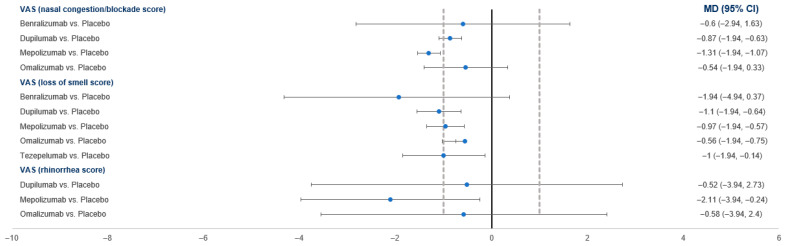
A forest plot showing the summary meta-analytic estimates (mean difference) from the placebo-focused comparisons with other biologic drugs regarding the visual analog scale (VAS) score across nasal congestion, loss of smell, and rhinorrhea domains.

Compared to the placebo, Benralizumab, dupilumab, and mepolizumab were associated with a significantly greater improvement in the nasal congestion/blockade score, while omalizumab showed no effect (Figure 5). In the loss of smell domain, benralizumab showed no effect, while other drugs (dupilumab, mepolizumab, omalizumab, and Tezepelumab) showed a greater improvement compared to the placebo. Regarding the rhinorrhea score, only mepolizumab showed a greater benefit compared to the placebo (MD = −2.11; 95% CI: −3.98, −0.24).

In the network analysis, mepolizumab was ranked the best in the nasal congestion/blockade (SUCRA = 69.6%) and rhinorrhea (SUCRA = 67.3%) domains, while Benralizumab was ranked the best in the loss of smell domain (SUCRA = 69.2%). The placebo was ranked the worst in all domains (Table 3).

#### 3.4.6. TNSS Score

Compared to the placebo, both CM310 and Omalizumab showed a significantly greater improvement (Figure 3). In the network analysis, CM310 (SUCRA = 80.7%) was ranked the best, followed by Omalizumab (SUCRA = 80.7%). The placebo was ranked the worst (SUCRA = 99.6%) (Table 3).

#### 3.4.7. UPSIT

Compared to the placebo, omalizumab showed no difference, while other drugs showed greater improvement, with dupilumab showing the highest benefit (MD = 12.14; 95% CI: 7.92, 16.38) (Figure 3). In the network analysis, dupilumab (SUCRA = 57%) was ranked the best followed by CM310 (SUCRA = 35.9%) and Mepolizumab (SUCRA = 54.6%), respectively. The placebo was ranked the worst (SUCRA = 90.3%) (Table 3).

#### 3.4.8. Time to First NP Surgery

**Figure 6:**
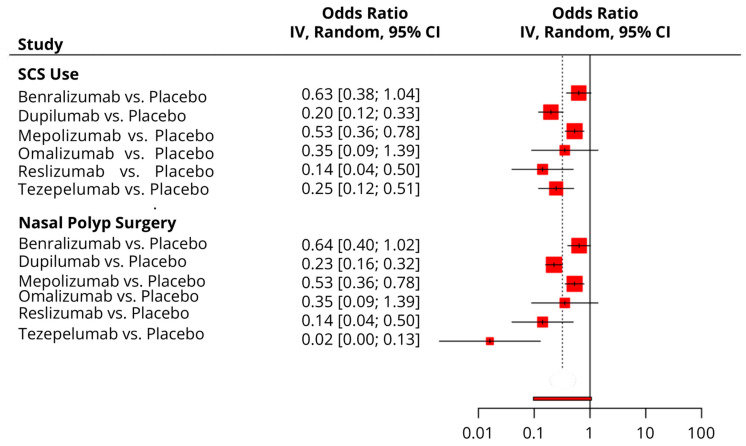
A forest plot showing the summary meta-analytic estimates (odds ratio) from the placebo-focused comparisons with other biologic drugs regarding the risk of systemic corticosteroid use and nasal polyp surgery post-biologic therapy.

Compared to the placebo, Benralizumab and Omalizumab showed no difference, while other drugs exhibited a significantly lower risk of NP surgery, especially Tezepelumab (OR = 0.02; 95% CI: 0.001, 0.13) (Figure 6). In the network analysis, Tezepelumab was ranked the best (SUCRA = 95.7%), followed by Reslizumab (SUCRA = 66.2%) and dupilumab (SUCRA = 62.6%), respectively. The placebo was ranked the worst (SUCRA = 90.2%) (Table 3).

#### 3.4.9. Time to First SCS Use

Compared to the placebo, Benralizumab and Omalizumab showed no difference, while other drugs exhibited a significantly lower risk of SCS use, especially Reslizumab (OR = 0.14; 95% CI: 0.04, 0.50) (Figure 6). In the network analysis, Reslizumab was ranked the best (SUCRA = 60.1%), followed by dupilumab (SUCRA = 45.7%) and Tezepelumab (SUCRA = 41.5%), respectively. The placebo was ranked the worst (SUCRA = 89.7%) (Table 3).

### 3.5. Responder Analysis

#### 3.5.1. MCID (≥8.9 Points Reduction in SNOT-22)

**Figure 7:**
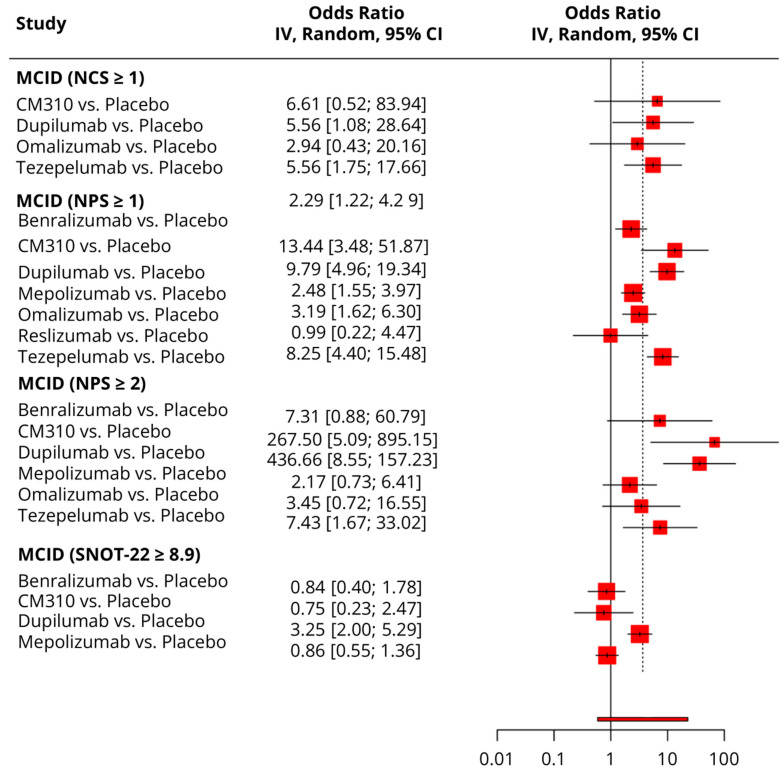
A forest plot showing the summary meta-analytic estimates (odds ratio) from the placebo-focused comparisons with other biologic drugs regarding responder analysis (minimal clinically important difference—MCID). The nasal polyp score (NPS) was assessed endoscopically as 0–4 per nostril and 0–8 in total (sum of left and right), where lower scores indicate smaller polyps; we prespecified patient-level responder thresholds of ≥1-point and ≥2-point reduction from baseline, consistent with the thresholds used in phase 3 trial responder analyses and supporting psychometric work. The nasal congestion score (NCS) was recorded on a 0–3 severity scale (0 none, 1 mild, 2 moderate, 3 severe), with a ≥1-point improvement from baseline prespecified as the within-patient responder threshold.

Compared to the placebo, dupilumab was the only drug to show a significantly greater odds of response (OR = 3.25; 95% CI: 2, 5.29). CM310, Benralizumab, and Mepolizumab showed no effect (Figure 7). In the network analysis, dupilumab was ranked the best (SUCRA = 98.4%), followed by Benralizumab (SUCRA = 29.1%) and Mepolizumab (SUCRA = 39.8%). The placebo was ranked the worst (SUCRA = 98.9%) (Table 3).

#### 3.5.2. MCID (≥1 Point Improvement in NCS)

Compared to the placebo, only dupilumab (OR = 5.56; 95% CI: 1.08, 28.64) and Tezepelumab (OR = 5.56; 95% CI: 1.75, 17.66) had significantly greater odds of response, while CM310 and Omalizumab showed no effect (Figure 7). In the network analysis, CM310 was ranked the best (SUCRA = 40.7%), followed by dupilumab (SUCRA = 26.7%) and Omalizumab (SUCRA = 33.2%), respectively. The placebo was ranked the worst (SUCRA = 77.3%) (Table 3).

#### 3.5.3. MCDI (≥1 Point Improvement in NPS)

Compared to the placebo, all drugs showed a significantly greater odds of response, except for reslizumab. CM310 showed the greatest benefit (OR = 13.44; 95% CI: 3.48, 51.87) (Figure 7). In the network analysis, CM310 was ranked the best (SUCRA = 60.4%), followed by dupilumab (SUCRA = 45%) and Omalizumab (SUCRA = 55.1%), respectively. Placebo was ranked the worst (SUCRA = 50.6%) (Table 3).

#### 3.5.4. MCID (≥2 Points Improvement in NPS)

Compared to the placebo, only CM310, dupilumab, and Tezepelumab showed a greater odd of response, while Benralizumab, Mepolizumab, and Omalizumab showed no effect (Figure 7). In the network analysis, CM310 was ranked the best (SUCRA = 64.7%), followed by dupilumab (SUCRA = 54.2%) and Tezepelumab (SUCRA = 36%), respectively. The placebo was ranked the worst (SUCRA = 85%) (Table 3).

### 3.6. Safety Analysis

Details regarding the safety profile and most common AEs by drug type can be found in Table 4.

**Figure 8:**
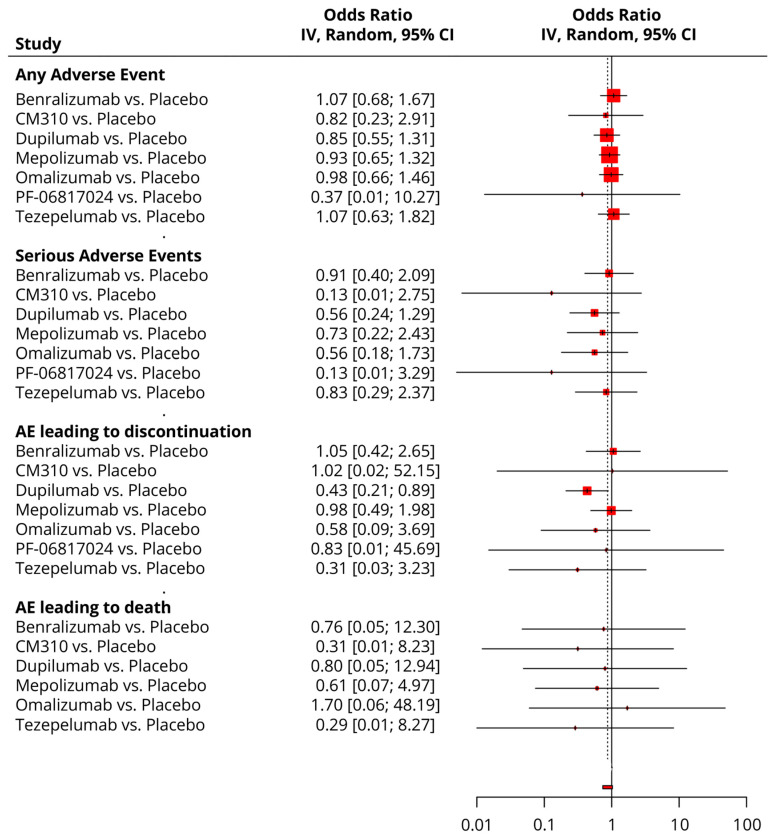
A forest plot showing the summary meta-analytic estimates (odds ratio) from the placebo-focused comparisons with other biologic drugs regarding the safety profile.

All drugs showed similar risk profiles to the placebo in terms of any or serious AEs, AEs leading to discontinuation, and AEs leading to death (Figure 8). In the network analysis, PF-06817024 was ranked the best in any AE (SUCRA = 58.5%) and SAE (SUCRA = 44.7%); Tezepelumab was ranked the best in AE leading to discontinuation (SUCRA = 33%); and CM310 was ranked the best in AE leading to death (SUCRA = 31.2%) (Table 3).

## 4. Discussion

This updated network meta-analysis synthesizes the current body of randomized evidence on biologic therapies for CRSwNP, incorporating data from 22 placebo-controlled RCTs, including two newly published trials [8,9]. By analyzing outcomes across a broad spectrum of efficacy domains—ranging from objective endoscopic and radiologic measures to patient-reported symptoms and responder-based thresholds—this study offers a robust comparative framework grounded in both direct and indirect evidence. Moreover, the incorporation of SUCRA-based treatment hierarchies and the use of a harmonized timepoint approach (LOCF) lend methodological rigor and clinical relevance to our findings.

### 4.1. Principal Findings

Our findings reaffirm the superior and consistent performance of dupilumab across the majority of evaluated outcomes, including NPS, NCS, SNOT-22 total score, and UPSIT, echoing prior conclusions from both trial-based and real-world meta-analyses [61,62,63]. Dupilumab was the only agent to consistently rank among the top three treatments across most domains, and was the only biologic associated with significantly greater odds of achieving clinically meaningful improvements in both the SNOT-22 and NCS responder analyses.

CM310, a newer IL-4/IL-13 pathway inhibitor, also demonstrated promising efficacy, particularly in CT score reduction and TNSS improvement, ranking first or second in several outcome domains. These findings complement recent real-world evidence suggesting emerging roles for newer agents beyond dupilumab [52]. Tezepelumab, though less consistently ranked, showed notable performance in delaying time to NP surgery, outperforming all other agents on this outcome—a finding that may hold clinical value in reducing the surgical burden for high-risk patients.

While omalizumab showed modest efficacy in nasal polyp size and TNSS, its performance was less compelling in VAS and SNOT-22 responder thresholds, aligning with the relatively attenuated treatment effects reported in previous meta-analyses [53,54]. Mepolizumab and benralizumab exhibited domain-specific efficacy—particularly for nasal congestion and loss of smell—but were not among the top-ranked treatments in most global or composite outcomes.

Importantly, our network meta-analysis integrates responder thresholds—such as MCID-based definitions for SNOT-22 and NPS—that are rarely reported in earlier NMAs, yet highly relevant to clinical decision-making [55,56]. This allows for a more granular and patient-centered understanding of benefit, as opposed to relying solely on average mean differences. For example, although mepolizumab did not show superiority in global SNOT-22, it ranked highest in the emotion and sleep subdomains, suggesting selective benefit in specific quality-of-life dimensions.

CRSwNP is driven predominantly by type-2 inflammation in which epithelial “alarmins” (e.g., TSLP/IL-33) activate dendritic cells and type-2 lymphocytes, leading to downstream IL-4/IL-13/IL-5 signaling that promotes IgE class-switching, eosinophilia, edema, mucus hypersecretion, and olfactory epithelial dysfunction [61]. Dupilumab blocks IL-4Rα and thereby inhibits both IL-4 and IL-13, broadly modulating epithelial barrier/goblet cell activity, mucus and edema, and neural/olfactory pathways—consistent with large, cross-domain effects (NPS, NCS) and especially strong gains in olfaction, along with reductions in surgery/OCS use [62]. Omalizumab neutralizes free IgE and down-regulates FcεRI on mast cells/basophils, attenuating allergen and S. aureus superantigen–driven inflammation; clinically, it improves congestion/symptoms and NPS, though structural effects can be smaller than with IL-4/13 blockade [63]. Mepolizumab (anti-IL-5) and benralizumab (anti-IL-5Rα; ADCC-mediated eosinophil depletion) directly reduce eosinophil survival/abundance, yielding pronounced effects on polyp burden (NPS) and surgery/OCS sparing, with more modest improvements in smell when epithelial IL-13 signaling remains active [64]. Tezepelumab (anti-TSLP) acts upstream, dampening multiple T2 effector pathways and showing broad, emerging benefits across NPS, NCS, and healthcare use outcomes in phase 3 trials [65]. Investigational IL-4Rα agents (e.g., CM310) may reproduce a dupilumab-like profile, whereas reslizumab (anti-IL-5) has a smaller evidence base with short-term NPS improvements in selected populations [66]. Taken together, these mechanisms align with our network findings: IL-4/13 blockade delivers the broadest—and often olfaction-leading—benefits; eosinophil-targeted agents excel on polyp size and surgery outcomes; and IgE/TSLP targeting provides domain-dependent gains that vary with patient endotype and upstream drivers.

### 4.2. Comparison with Prior Reviews

The findings of this updated NMA are generally concordant with earlier syntheses showing the dominance of dupilumab in reducing polyp burden and improving symptoms [54,67,68]. However, this work expands upon prior reviews in several key respects. First, it incorporates two recent RCTs that were not included in past analyses, thereby enhancing statistical power and generalizability. Second, while earlier reviews often pooled biologics together or compared them narratively [60,69,70], our study uses a fully connected NMA model to enable formal ranking and SUCRA-based inferences.

Moreover, previous reviews have often been limited by the heterogeneity in outcome measures, variable follow-up durations, and inconsistent timepoints for reporting [71]. By using a LOCF strategy, we address this challenge and enhance the temporal comparability across trials. Our results also offer a more detailed exploration of domain-specific effects—for instance, identifying Omalizumab as the most effective agent in the ear/facial pain subdomain of SNOT-22, a nuance not captured in earlier syntheses [53,70].

Additionally, the current study provides an updated safety comparison, showing no significant differences in the risk of adverse events between any of the biologics and placebo, consistent with prior safety syntheses [52,67]. Of note, PF-06817024 ranked favorably in AE profiles, although efficacy data for this agent were sparse.

### 4.3. Clinical Implications

The collective evidence reinforces the role of dupilumab as the reference standard in biologic treatment for CRSwNP, particularly in patients with recalcitrant disease or those seeking improvements across multiple symptom dimensions. CM310 and tezepelumab may represent viable alternatives in patients with partial responses or contraindications to dupilumab, especially in settings where CT findings or the need for surgery are primary concerns. Notably, our findings suggest that no single biologic offers uniform superiority across all domains—underscoring the need for individualized, endotype-driven therapy selection, as previously emphasized in guideline-based and mechanistic reviews [54,67,69].

Furthermore, the differential performance in responder thresholds suggests that traditional metrics such as mean differences may underestimate the real-world relevance of biologic therapy, particularly in shared decision-making scenarios. Clinicians may consider prioritizing agents based not only on global efficacy but also on patient-specific goals (e.g., sleep quality, smell recovery), particularly when multiple options are available.

### 4.4. Clinical Selection and Access (Practical Considerations)

Our network quantifies comparative efficacy and safety, but the choice of biologic in practice also depends on patient priorities, endotype/phenotype, comorbid asthma, prior surgery/OCS exposure, dosing logistics, local reimbursement criteria, and patient preference. In general, IL-4Rα blockade (dupilumab; investigational CM310) offers broad, cross-domain benefits and particularly strong gains in olfaction, making it attractive when the restoration of smell and global symptom relief are priority outcomes. Anti-IL-5/IL-5R agents (mepolizumab, benralizumab) directly target eosinophilic inflammation and often excel for polyp burden and surgery/OCS sparing, which may suit patients with high eosinophil counts or recurrent post-surgical disease. Anti-IgE therapy (omalizumab) is most compelling in IgE-driven/atopic disease and improves congestion/symptoms with meaningful effects on NPS in appropriate candidates. Anti-TSLP (tezepelumab) acts upstream and shows a broadening efficacy profile; its role will likely expand as real-world experience accumulates. Importantly, reimbursement policies—which vary by health system and may specify biomarker thresholds, prior surgery, OCS exposure, or other criteria—can channel prescriptions toward certain agents despite overlapping efficacy. Accordingly, we recommend aligning the choice of biologic with (1) the dominant clinical goals (e.g., olfaction vs. polyp size vs. OCS/surgery reduction), (2) biologic plausibility/endotype (e.g., eosinophilia, IgE level, comorbid asthma), (3) safety/tolerability and dosing cadence, and (4) access and patient preference, recognizing that several agents can be effective and that switching may be appropriate when goals are unmet.

There is no universally agreed stopping date for biologics in CRSwNP. Expert guidance favors periodic response assessment to inform continuation or change rather than a fixed duration: an early review at ~16 weeks (to stop or switch in non-responders), a comprehensive reassessment at 6–12 months, and annual follow-up thereafter. In well-controlled patients, dose-spacing (e.g., extending dupilumab dosing to every 4 weeks) may sustain control, whereas abrupt discontinuation has been associated with recurrence in some reports; decisions should be individualized and revisited with shared decision-making. Given the heterogeneity of disease courses and payer policies, we advise aligning duration with the clinical goals achieved (polyp size, congestion, olfaction, OCS/surgery sparing), tolerability, and access, with a low threshold to de-escalate or switch if targets are not met.

### 4.5. Limitations

This analysis is not without limitations. Although we included the most recent RCTs, the lack of head-to-head trials remains a key barrier to definitive comparative claims—an issue highlighted in all prior reviews [53,68,70]. Additionally, while LOCF mitigates inconsistencies in follow-up timing, it may obscure dynamic changes over time and potentially underrepresent delayed treatment effects. Furthermore, subgroup effects (e.g., based on comorbid asthma or baseline eosinophil counts) could not be explored due to insufficient data granularity, limiting precision in endotype-specific recommendations—a gap similarly noted by others [52,71].

Additionally, our analysis cannot specify stopping rules; existing recommendations prioritize reassessment-based continuation rather than fixed durations, and evidence on de-escalation vs. discontinuation remains limited. Finally, while our focus on placebo-controlled comparisons enhances internal validity, it precludes conclusions about the relative superiority of one biologic over another—a tradeoff also observed in previous indirect comparisons [55,67].

### 4.6. Future Directions

Given the domain-specific benefits identified for certain agents, future research should focus on biomarker-based personalization and prospective head-to-head trials. Greater emphasis on harmonized outcome reporting, the standardization of MCID thresholds, and the integration of patient-centered domains will improve comparability and clinical applicability. In addition, the utility of combination strategies or stepwise switching protocols, particularly in patients with overlapping severe asthma, remains to be explored [56].

## 5. Conclusions

This updated network meta-analysis, encompassing 22 placebo-controlled RCTs and over 4000 patients with CRSwNP, provides a comprehensive comparative evaluation of biologic therapies across multiple efficacy and safety domains. Dupilumab consistently emerged as the most effective agent across global and domain-specific outcomes, including polyp size, symptom scores, and quality-of-life indices. CM310 and Tezepelumab also demonstrated notable efficacy in specific domains such as radiologic outcomes and surgical delay. Importantly, all biologics exhibited comparable safety profiles to placebo.

These findings reinforce the role of biologic therapies—particularly dupilumab—as an essential component in the treatment algorithm for patients with severe or refractory CRSwNP. However, no single agent showed uniform superiority across all outcomes, underscoring the need for personalized, endotype-driven treatment strategies. Future head-to-head trials, long-term follow-up studies, and standardized outcome reporting will be critical to optimizing therapeutic selection and improving patient-centered care in CRSwNP.

## Figures and Tables

**Table 1:** Descriptive summary of randomized controlled trials assessing the efficacy of different biological therapy agents in patients with CRSwNP.

Trial Name	Author (YOP)	Country	YOI	Population	Allocation		**Sample**		
					Intervention	**Control**	**Intervention**	**Control**	**Total**
BREATHE Phase III Trials (post hoc) NCT01287039/NCT01285323	Weinstein (2019) [16]	Trial 1: 128 centers Trial 2: 104 centers (Asia, Australia, North America, South America, South Africa, and Europe)	Trial 1 (Apr 2011–Mar 2014) Trial 2 (Mar 2011–Apr 2014)	Self-reported CRSwNP (with or without Aspirin sensitivity)	Reslizumab (3 mg/kg, IV) every 4 wks for 52 wks	Placebo	78	72	150
Phase IIIb Trial NCT03170271	Canonica (2022) [58]	221 clinical research centers	Jul 2017–Sept 2019	CRSwNP of any severity	Benralizumab (30 mg every 8 wks; first 3 doses every 4 wks)	Placebo	96	57	153
CROWNS-1 Phase II Trial NCT04805398	Zhang (2023) [59]	19 hospitals in China	Apr 2021–Mar 2022	CRSwNP patients had received SCS treatment within 2 years prior to the run-in period, or have contraindicated or intolerant to SCS treatment or have undergone nasal polyp surgery 6 months before the run-in period, to have an NPS of at least 5 points (at least 2 points for each nostril), and to have moderate or severe nasal congestion with a weekly average nasal congestion score (NCS) of 2 or 3 points and any other symptoms such as loss of smell or rhinorrhea.	CM310 (subcutaneous 300 mg; every 2 wks) for 16 wks	Placebo	28	28	56
NAVIGATORY Phase III Trial (subgroup) NCT03347279	Laidlaw (2023) [60]	297 sites in 18 countries	Nov 2017–Sep 2020	CRSwNP of any severity	Tezepelumab (subcutaneous 210 mg every 4 wks) for 52 wks	Placebo	62	56	118
Phase I Trial	Gevaert (2006) [17]	2 centers	-	CRSwNP [massive bilateral NP grade 3–4 or recurrent NP after surgery]	Reslizumab (single IV infusion at 1 mg/kg)	Placebo	8	8	24
					Reslizumab (single IV infusion at 3 mg/kg)	Placebo	8		
-	Gevaert (2013) [18]	Belgium	Jan 2007–Oct 2008	CRSwNP [according to European Position Paper on Rhinosinusitis and Nasal Polyps Guidelines]	Omalizumab (4–8 subcutaneous doses; once every 2 wks) for 16 wks	Placebo	15	8	23
Phase II Trial NCT02772419	Takabayashi (2021) [18]	Multicenter, Japan	May 2016–May 2017	Eosinophilic CRSwNP [bilateral NPS of 3 with a score ≥ 1 in each nostril]	Benralizumab (single dose 30 mg)	Placebo	22	11	56
					Benralizumab (3 doses of 30 mg; once every 4 weeks)		23		
-	Wahba (2019) [19]	Egypt	Jan 2015–May 2018	CRSwNP [according to the criteria defined by the rhinosinusitis Task Force]	Omalizumab (0.016 mg/kg/IgE (IU/mL)); once every 2 wks	Conventional therapy (ABs + corticosteroid)	43	43	86
NCT03450083	Tversky (2021) [20]	USA	Jan 2018–Dec 2019	Severe CRSwNP (average bilateral NPS ≥ 5), eosinophil count of 300/Ml or greater, refractory symptoms despite prior surgical or endoscopic NP removal, and at least one OCS course over the previous 12 months	Benralizumab (30 mg SC) every 4 wks for 20 wks	Placebo	12	12	24
-	Boiko (2023) [11]	Russia	Jan 2019–Nov 2022	CRSwNP [with the need for SCS over the past 2 years and deteriorating QoL with reduction in olfaction]	Dupilumab (300 mg SC every 2 wks for 24 wks)	Reslizumab (3 mg/kh IV once every 4 wks for 24 wks)	10	9	19
CRT110178	Gevaert (2011) [21]	Belgium	-	Severe CRSwNP (grade 3–4 or recurrent after surgery) refractory to CS	Mepolizumab (750 mg, 2 IV injections 28 days apart)	Placebo	20	10	30
NCT01362244	Bachert (2017) [22]	6 centers (Belgium, The Netherlands, and UK)	May 2009–Dec 2014	Severe, recurrent, bilateral NP and required NP surgery with NPS ≥ 3 in 1 nostril and VAS symptom score > 7	Mepolizumab (6 doses of 750 mg IV, once every 4 wks)	Placebo	54	51	105
Phase I Trial NCT02743871	Danto (2024) [23]	20 centers in USA	-	CRSwNP (not defined)	PF-06817024 (single dose, 30 mg IV)	Placebo	11	9	20
Post hoc of 2 trials [18,21]	De Schryver (2017) [24]	Belgium	-	Same criteria as ID [9,25]	Omalizumab	Placebo	15	8	23
					Mepolizumab	Placebo	20	10	20
OSTRO Phase III Trial NCT03401229	Bachert (2022) [26]—Main Results	102 sites in Europe and the US	Jan 2018–Jul 2020	CRSwNP [Bilateral NP with NPS ≥ 5 “with unilateral score ≥ 2” despite maintenance treatment with INCS for at least 4 weeks before enrollment and a history of SCS use and/or NP surgery]	Benralizumab (30 mg SC; every 4 wks for the 1st 3 doses and every 8 wks after)	Placebo	207	203	410
	Emson (2024) [27]—Post hoc								
POLYP 1-2 & OLE Phase III Trials NCT03280550, NCT03280537, NCT03478930	Gevaert (2020) [28]–POLYP 1/2 (Main Results)	North America and Europe	Nov 2017–Mar 2019	CRSwNP with inadequate response to INCS for 4 weeks before screening with NPS ≥ 5 (≥2 for each nostril)	Omalizumab (75–600 mg (based on total IgE level and body weight) SC injection every 2–4 wks) for 24 wks	Placebo	72	66	138
					Omalizumab (75–600 mg (based on total IgE level and body weight) SC injection every 2–4 wks) for 52 wks	Placebo	62	65	127
	Gevaert (2022) [29]—OLE Trial				Omalizumab (75–600 mg (based on total IgE level and body weight) SC injection every 2–4 wks) for 52 wks	Placebo (received omalizumab for 28 wks)	124	125	249
	Damask (2022) [30]—POLYP 1/2 (Post hoc)								
	Han (2022) [31]—POLYP 1/2 (Post hoc)								
	Meltzer (2024) [32]—POLYP 1/2 (Post hoc)								
	Gevaert (2024) [33]—POLYP 1/2 & OLE								
QUEST Phase III Trial NCT02414854	Hopkins (2022) [34]—Subgroup analysis	Europe, Western Countries, Asia, and Latin America	May 2015–Sep 2016	CRSwNP [History at baseline]	Dupilumab (300 mg SC every 2 wks for 52 wks)	Placebo	123	70	193
	Bourdin (2022) [35]—Post hoc				Dupilumab (200–300 mg SC every 2 wks for 52 wks)	Placebo	66	43	107
	Maspero (2023) [36]—Post hoc								
SINUS 24 & 52 Phase III Trials	Bachert (2019) [25]—Main Results	67 centers in 13 countries	Dec 2016–Aug 2017	Bilateral NP and symptoms of chronic rhinosinusitis despite INCS therapy before enrollment and had received SCS in the previous 2 years or previous NP surgery	Dupilumab (SC 300 mg every 2 wks) for 24 wks	Placebo	143	133	276
		117 centers in 14 countries	Nov 2016–Aug 2017		Dupilumab (300 mg SC, once every 2 wks) for 52 wks		150	153	448
					Dupilumab (300 mg SC, once every 4 wks) for 52 weeks		145		
	Desrosiers (2021) [37]—Post hoc	--------Same as Original Trial--------			------------------------------- Same as Original Trial --------------------------------				
	Mullol (2022) [38]—Post hoc								
	Fujieda (2022) [39]—Post hoc								
	Lee (2022) [40]—Post hoc								
	Bachert (2022) [41]—Post hoc								
	Chuang (2022) [31]—Post hoc								
	Bachert (2023) [42]—Post hoc								
	Gurnell (2024) [43]—Post hoc								
SNAPSE & OLE (treatment-free) Phase III Trials NCT03085797	Han (2021) [44]—Main Results	93 centers in 11 countries	May 2017–Dec 2018	CRSwNP [recurrent, bilateral, refractory, severe NP symptoms with nasal obstruction VAS score >5 and were eligible for repeat NP surgery with NPS ≥ 5]	Mepolizumab (100 mg SC every 4 wks for 52 wks)	Placebo	206	201	407
	Hopkins (2023) [45]—Post hoc								
	Fokkens (2023) [57]—Post hoc								
	Chupp (2023) [46]—Post hoc								
	Desrosiers (2024) [47]—OLE Trial						69	65	134
Phase IIa Trial NCT01920893	Bachert (2016) [48]—Main Results	13 sites in the US and Europe	Aug 2013–Aug 2014	CRSwNP [bilateral NP and chronic symptoms of rhinosinusitis despite INCS treatment ≥ 2 months, and ≥ 2 rhinosinusitis symptoms (nasal obstruction, nasal discharge, facial pain/pressure, reduction/loss of smell]	Dupilumab (600 SC mg loading dose followed by 300 mg weekly)	Placebo	30	30	60
	Bachert (2019) [25]—Post hoc						16	19	35
	Jonstam (2019) [49]—Post hoc								
	Bachert (2020) [50]—Post hoc								
	Bachert (2020) [51]—Post hoc								
WAYPOINT NCT04851964	Lipworth (2025)—Main [9]	112 sites in 10 countries (Canada, China, Denmark, Germany, Hungary, Japan, Poland, Spain, UK, and USA)	Apr 2021–Aug 2023	Patients with physician-diagnosed CRSwNP for at least 12 months with NPS of at least 5, NCS of at least 2, and SNOT-22 of at least 30	Tezepelumab (210 mg) every 4 weeks for 52 weeks	Placebo	203	205	408
MERIT NCT04607005	Fujieda (2024)—Main [8]	60 centers (Japan, China, Russia)	Feb 2021–Mar 2022	Patients with blood eosinophil count >2%, endoscopic NPS of at least 5, nasal obstruction VAS score of at least 5, and sinonasal symptoms of at least 2, and either prior NPS or SCS use	Mepolizumab (100 mg SC) every 4 weeks for 52 weeks	Placebo	84	85	169

YOI: Year of Investigation; YOP: Year of Publication; US: United States; CRSwNP: Chronic Rhinosinusitis with Nasal Polyps; NP: Nasal Polyp; SCS: Systemic Corticosteroid; INCS: Intranasal Corticosteroid; SC: Subcutaneous; IV: Intravenous; NPS: Nasal Polyp Score; wk: Week; OLE: Open-Label Extension.

**Table 2:** Descriptive summary of comorbid asthma, history of NPS surgery, disease duration, and patients’ characteristics at baseline among the included randomized trials of biological therapy in CRSwNP.

Trial Name	Author (YOP)	Allocation		Comorbid Asthma (%)			Prior NP Surgery (%)		CRSwNP Duration (yr); M (SD)		Gender [M/F]			**Age [Mean/SD]**			**FU [wk]**
		Intervention	Control	Intervention	Control	Definition	Intervention	Control	Intervention	Control	Intervention	Control	**Total**	**Intervention**	**Control**	**Total**	
BREATHE Phase III Trials (post hoc) NCT01287039/NCT01285323	Weinstein (2019) [16]	Reslizumab (3 mg/kg, IV) every 4 wks for 52 wks	Placebo	100	100	Inadequately controlled eosinophilic asthma (≥400 cells/mL) on at least medium-dose ICS	-	-	-	-	-	-	57/93	-	-	51.1 (11.2)	52
Phase IIIb Trial NCT03170271	Canonica (2022) [58]	Benralizumab (30 mg every 8 wks; first 3 doses every 4 wks)	Placebo	100	100	Severe, eosinophilic asthma who had experienced ≥2 prior-year exacerbations despite high-dosage inhaled corticosteroid plus additional controller(s)	71.9	71.9	-	-	43/53	33/24	76/77	53.1 (12.3)	52.6 (11.1)	53 (11.5)	24
CROWNS-1 Phase II Trial NCT04805398	Zhang (2023) [59]	CM310 (subcutaneous 300 mg; every 2 wks) for 16 wks	Placebo	64	68	-	57	68	8.9 (9.3)	8.9 (7.6)	18/10	14/14	32/24	48.8 (12.2)	46.4 (12.5)	47.6 (12.3)	16
NAVIGATORY Phase III Trial (subgroup) NCT03347279	Laidlaw (2023) [60]	Tezepelumab (subcutaneous 210 mg every 4 wks) for 52 wks	Placebo	100	100	Physician-diagnosed asthma, for patients who had received medium- or high-dose inhaled glucocorticoids (daily dose of ≥500 μg of fluticasone propionate or equivalent) for at least 12 months before screening and at least one additional controller medication, with or without oral glucocorticoids, for at least 3 months	-	-	-	-	25/37	28/28	53/65	51.2 (13.3)	50.4 (12.6)	50.8 (12.9)	52
Phase I Trial	Gevaert (2006) [17]	Reslizumab (single IV infusion at 1 mg/kg)	Placebo	87.5	75	Asthma history	25	50	-	-	6/2	6/2	16/8	43.6 (12.6)	48 (12.1)	-	36
		Reslizumab (single IV infusion at 3 mg/kg)	Placebo	62.5			75		-	-	4/4			48.5 (18.1)			36
-	Gevaert (2013) [18]	Omalizumab (4–8 subcutaneous doses; once every 2 wks) for 16 wks	Placebo	100	100	Severe allergic asthma [based on Global Initiative for Asthma Guidelines and diagnosed with respiratory physicians]	87	75	-	-	12/3	4/4	16/7	50 (14.4)	45 (14.2)	47.3 (14.3)	16
Phase II Trial NCT02772419	Takabayashi (2021) [18]	Benralizumab (single dose 30 mg)	Placebo	81.8	90.9	Comorbid asthma	59.1	72.7	-	-	11/11	7/4	30/26	54 (10.8)	53.3 (14.8)	53.7 (12.9)	12
		Benralizumab (3 doses of 30 mg; once every 4 weeks)		82.6			65.2		-	-	12/11			53 (12.3)			12
-	Wahba (2019) [19]	Omalizumab (0.016 mg/kg/IgE (IU/mL)); once every 2 wks	Conventional therapy (ABs + corticosteroid)	-	-	-	-	-	9.2 (3.6) mo	9.8 (2.8) mo	28/15	26/17	54/32	37 (11.7)	37.5 (10.2)	-	16
NCT03450083	Tversky (2021) [20]	Benralizumab (30 mg SC) every 4 wks for 20 wks	Placebo	83	100	Asthma history	number: 3 (2.3) *	2.3 (1.6) *	10 (5.1)	11.1 (14.4)	7/5	7/5	14/10	49.8 (12.1)	50.8 (13.1)	-	20
-	Boiko (2023) [11]	Dupilumab (300 mg SC every 2 wks for 24 wks)	Reslizumab (3 mg/kh IV once every 4 wks for 24 wks)	100	100	Severe eosinophilic asthma	-	-	(6–29)	(6–28)	3/7	3/6	6/13	28–69	29–58	-	24
CRT110178	Gevaert (2011) [21]	Mepolizumab (750 mg, 2 IV injections 28 days apart)	Placebo	50	30	Asthma history	75	80	10.5 (5.6)	14.3 (8.23)	14/6	8/2	22/8	50.05 (8.86)	45.9 (11.43)	-	48
NCT01362244	Bachert (2017) [22]	Mepolizumab (6 doses of 750 mg IV, once every 4 wks)	Placebo	81	75	Asthma history	-	-	-	-	34/17	41/13	75/30	51 (11)	50 (10)	-	25
Phase I Trial NCT02743871	Danto (2024) [23]	PF-06817024 (single dose, 30 mg IV)	Placebo	-	-	-	-	-	-	-	8/3	5/4	13/7	54.4 (6.2)	42.8 (10.7)	-	3
Post hoc of 2 trials [ID [9,17]	De Schryver (2017) [24]	Omalizumab	Placebo	100	100	-	-	-	-	-	12/3	4/4	16/7	50 (14.4)	45 (14.2)	-	8
		Mepolizumab	Placebo	50	30						14/6	8/2	22/8	50.05 (8.86)	45.9 (11.43)		
OSTRO Phase III Trial NCT03401229	Bachert (2022) [26]—Main Results	Benralizumab (30 mg SC; every 4 wks for the 1st 3 doses and every 8 wks after)	Placebo	68.6	67	Comorbid asthma	72.9	73.4	6.93 (6.45)	6.95 (5.46)	142/65	121/82	263/147	50.1 (12.4)	50.2 (13.9)	-	40 (extended to 56)
	Emson (2024) [27]—Post hoc																
POLYP 1-2 & OLE Phase III Trials NCT03280550, NCT03280537, NCT03478930	Gevaert (2020) [28]—POLYP 1/2 (Main Results)	Omalizumab (75–600 mg (based on total IgE level and body weight) SC injection every 2–4 wks) for 24 wks	Placebo	58.3	48.5	Comorbid asthma	54.2	60.6	-	-	47/25	41/25	88/50	50 (14.5)	52.2 (11.6)	-	24
		Omalizumab (75–600 mg (based on total IgE level and body weight) SC injection every 2–4 wks) for 52 wks	Placebo	61.3	60		62.9	61.5			39/23	44/21	83/44	49 (1.9)	51 (12)		24
	Gevaert (2022) [29]—OLE Trial	Omalizumab (75–600 mg (based on total IgE level and body weight) SC injection every 2–4 wks) for 52 wks	Placebo (received omalizumab for 28 wks)	59.7	54.4		56.5	61.6			79/45	81/44	160/89	50 (13.1)	51.5 (11.8)		52
	Damask (2022) [30]—POLYP 1/2 (Post hoc)																24–52
	Han (2022) [31]—POLYP 1/2 (Post hoc)																
	Meltzer (2024) [32]—POLYP 1/2 (Post hoc)																
	Gevaert (2024) [33]—POLYP 1/2 & OLE																
QUEST Phase III Trial NCT02414854	Hopkins (2022) [34]—Subgroup analysis	Dupilumab (300 mg SC every 2 wks for 52 wks)	Placebo	100	100	Uncontrolled moderate-to-severe asthma	-	-	19.8	-	48/75	22/48	70/123	52.7 (13.5)	49.6 (11.8)	51.6 (13)	52
	Bourdin (2022) [35]—Post hoc	Dupilumab (200–300 mg SC every 2 wks for 52 wks)	Placebo	100	100	Type 2 asthma (with high-dose ICS)			-		-	-	-	-	-	-	
	Maspero (2023) [36]—Post hoc																
SINUS 24 & 52 Phase III Trials	Bachert (2019) [25]—Main Results	Dupilumab (SC 300 mg every 2 wks) for 24 wks	Placebo	59	57	Asthma	69	74	11.42 (9.69)	10.77 (8.57)	88/55	70/63	158/118	52 (13.9)	50 (14.6)	-	e+Y36---------- Sam
		Dupilumab (300 mg SC, once every 2 wks) for 52 wks		57	59		59	58	11.28 (10.38)	10.88 (9.4)0	97/53	95/58	279/169	51 (14.2)	53 (14.4)		
		Dupilumab (300 mg SC, once every 4 wks) for 52 weeks		63			59		10.67 (9.12)		87/58			53 (14.2)			
	Desrosiers (2021) [37]—Post hoc	--------Same like Original Trial-------		----------------------------------------------- Same like Original Trial --------------------------------------------------------------													
	Mullol (2022) [38]—Post hoc																
	Fujieda (2022) [39]—Post hoc																
	Lee (2022) [40]—Post hoc																
	Bachert (2022) [41]—Post hoc																
	Chuang (2022) [31]—Post hoc																
	Bachert (2023) [42]—Post hoc																
	Gurnell (2024) [43]—Post hoc																
SNAPSE & OLE (treatment-free) Phase III Trials NCT03085797	Han (2021) [44]—Main Results	Mepolizumab (100 mg SC every 4 wks for 52 wks)	Placebo	68	74	Asthma	100	100	11.4 (8.5)	11.5 (8.3)	139/67	125/76	264/143	48.6 (13.6)	48.9 (12.5)	-	52
	Hopkins (2023) [45]—Post hoc																
	Fokkens (2023) [57]—Post hoc																
	Chupp (2023) [46]—Post hoc																
	Desrosiers (2024) [47]—OLE Trial			-	-	-	-	-	-	-	-	-	-	-	-	-	76
Phase IIa Trial NCT01920893	Bachert (2016) [48]—Main Results	Dupilumab (600 SC mg loading dose followed by 300 mg weekly)	Placebo	53.3	63.3	Comorbid asthma	53.3	63.3	7.6 (6.1)	11.5 (8.7)	16/14	18/12	34/26	49.3 (9.1)	47.4 (9.8)	-	16
	Bachert (2019) [25]—Post hoc			31.3	42.1	Aspirin-sensitive asthma	-	-	11.32 (8.93)	8.95 (6.33)	7/9	7/12	14/21	51.4 (7.6)	47.7 (9.9)		
	Jonstam (2019) [49]—Post hoc																
	Bachert (2020) [50]—Post hoc																
	Bachert (2020) [51]—Post hoc																
WAYPOINT NCT04851964	Lipworth (2025)—Main [9]	Tezepelumab (210 mg) every 4 weeks for 52 weeks	Placebo	60.1	61.5	Coexisting asthma	70.9	71.7	12.71 (10.43)	12.8 (10.34)	126/77	140/65	266/142	50.1 (13.6)	49.4 (13.7)	49.7 (13.6)	52
MERIT NCT04607005	Fujieda (2024)—Main [8]	Mepolizumab (100 mg SC) every 4 weeks for 52 weeks	Placebo	79	79	Concurrent asthma	65	64	11.9 (9.09)	10.9 (9.08)	53/31	56/29	109/60	52 (10.5)	52 (13.2)	-	52

* data are provided for the mean number of NP surgeries performed and not whether or not NP surgery had been performed before. YOP: Year of Publication; CRSwNP: Chronic Rhinosinusitis with Nasal Polyps; NP: Nasal Polyp; SC: Subcutaneous; IV: Intravenous; wk: Week; yr: Year; FU: Follow-up; OLE: Open-Label Extension.

**Table 3:** A summary of the SUCRA-based rankings of biologic drugs in CRSwNP across all measured outcomes.

Rank	Best	2nd Rank	3rd Rank	4th Rank	5th Rank	6th Rank	7th Rank	Worst
Any AE	PF-06817024	CM310	Dupilumab	Placebo	Mepolizumab	Omalizumab	Benralizumab	PF-06817024
SAE	PF-06817024	CM310	Dupilumab	Dupilumab	Dupilumab	Placebo	Placebo	Benralizumab
AE leading to discontinuation	Tezepelumab	Dupilumab	Dupilumab	Placebo	Placebo	Placebo	Benralizumab	PF-06817024
AE leading to death	CM310	Tezepelumab	Mepolizumab	Placebo	Placebo	Omalizumab	-	Omalizumab
MCID (NCS ≥ 1)	CM310	Dupilumab	Dupilumab	Omalizumab	-	-	-	Placebo
MCID (NPS ≥ 2)	CM310	Dupilumab	Tezepelumab	Tezepelumab	Omalizumab	Mepolizumab	-	Placebo
MCID (NPS ≥ 1)	CM310	Dupilumab	Dupilumab	Omalizumab	Mepolizumab	Benralizumab	Placebo	Placebo
MCID (SNOT-22 ≥ 8.9)	Dupilumab	Benralizumab	Benralizumab	Mepolizumab	-	-	-	Placebo
SCS Use	Reslizumab	Dupilumab	Tezepelumab	Mepolizumab	Mepolizumab	Benralizumab	-	Placebo
Nasal Polyp Surgery	Tezepelumab	Reslizumab	Dupilumab	Mepolizumab	Mepolizumab	Benralizumab	-	Placebo
Endoscopic NPS	Dupilumab	Tezepelumab	Tezepelumab	Omalizumab	Mepolizumab	Benralizumab	Placebo	Reslizumab
NCS	Dupilumab	Tezepelumab	CM310	Omalizumab	-	-	-	Placebo
Lund-Mackay CT score	CM310	Dupilumab	Tezepelumab	Omalizumab	Mepolizumab	Benralizumab	-	Placebo
UPSIT score	Dupilumab	CM310	Mepolizumab	Omalizumab	-	-	-	Placebo
TNSS score	CM310	Omalizumab	-	-	-	-	-	Placebo
QoL (EQ-5D VAS)	Dupilumab	Mepolizumab	-	-	-	-	-	Placebo
SNOT-22 (total score)	Dupilumab	Dupilumab	Mepolizumab	Mepolizumab	Omalizumab	Benralizumab	-	Placebo
SNOT-22 (emotion score)	Mepolizumab	Omalizumab	Dupilumab	Tezepelumab	-	-	-	Placebo
SNOT-22 (function score)	Dupilumab	Tezepelumab	-	-	-	-	-	Placebo
SNOT-22 (sleep score)	Mepolizumab	Dupilumab	Omalizumab	Tezepelumab	-	-	-	Placebo
SNOT-22 (ear/facial pain score)	Omalizumab	Mepolizumab	Dupilumab	Tezepelumab	-	-	-	Placebo
SNOT-22 (nasal symptom score)	Omalizumab	Mepolizumab	Dupilumab	Tezepelumab	-	-	-	Placebo
VAS (nasal congestion/blockade score)	Mepolizumab	Dupilumab	Dupilumab	Placebo	-	-	-	Placebo
VAS (loss of smell score)	Benralizumab	Dupilumab	Mepolizumab	Mepolizumab	Omalizumab	-	-	Placebo
VAS (rhinorrhea score)	Mepolizumab	Omalizumab	Placebo	-	-	-	-	Placebo

CRSwNP: chronic rhinosinusitis with nasal polyps; AE: adverse event; SAE: serious adverse event; MCID: minimal clinically important difference; SCS; systemic corticosteroid; NCS: nasal congestion score; NPS: nasal polyp score; SNOT: Sino-Nasal Outcome Test; CT: computed tomography; QoL: quality of life; VAS: visual analog scale.

**Table 4:** A complete list of complications associated with biological therapy in patients with chronic rhinosinusitis with nasal polyps.

	Mepolizumab		Omalizumab		Dupiluzumab		Benralizumab		Reslizumab		Tezepelumab		CM310		**PF-06814024**		**Placebo**	
	%	95%CI	%	95%CI	%	95%CI	%	95%CI	%	95%CI	%	95%CI	%	95%CI	**%**	**95%CI**	**%**	**95%CI**
Acute sinusitis	6.3	3–9.6	2	0–5	-	-	-	-	-	-	-	-	-	-	-	-	6.6	3.2–10
Allergic reaction	6.5	1.8–11.2	7	6–19	-	-	-	-	-	-	-	-	-	-	-	-	4.26	0–14.5
Arterial thrombotic event	-	-	1	1–2	-	-	-	-	-	-	-	-	-	-	-	-	0.4	0.006–1.4
Arthralgia	6.1	3.2–9.1	2	1–4	-	-	-	-	-	-	3.4	1–6	-	-	-	-	2.18	1.2–4.5
Asthma (new-onset)	1.9	0.4–3.3	-	-	2	1–5	9	5–13	-	-	0.5	0–1.5	2	1–6	-	-	7.58	5.7–12.8
Asthma (exacerbation)	-	-	8	0–16	-	-	3	3–8	-	-	-	-	-	-	-	-	4.2	1.1–7.2
Back pain	3.8	0–7.5	4	1–7	10	1–21	-	-	-	-	4.9	1.9–7.9	-	-	18	5–41	4.7	1.8–7.6
Bronchitis	6.9	0–14.9	-	-	3	1–10	-	-	-	-	-	-	-	-	-	-	4.07	3.6–10
Common cold	25	6–44	53	28–79	-	-	6	2–10	-	-	-	-	-	-	-	-	7.3	1.4–19
Cough	3.7	1.4–6	-	-	2	1–6	-	-	-	-	-	-	-	-	18	5–41	6.1	3.4–8.9
COVID-19	-	-	-	-	-	-	-	-	-	-	23.2	17.4–29	-	-	-	-	18.97	-
CRSwNP	-	-	-	-	-	-	-	-	-	-	5.4	2.3–8.5	-	-	-	-	22.93	-
Disk herniation (pre-existing)	5	0–15	-	-	-	-	-	-	-	-	-	-	-	-	-	-	4.5	1.7–16.9
Diverticulitis (pre-existing)	5	0–15	-	-	-	-	-	-	-	-	-	-	-	-	-	-	4.5	1.7–16.9
Dizziness	2.4	0–5.6	1	1–2	10	1–21	-	-	-	-	-	-	-	-	4	3–15	4.5	0.6–5.7
Dyspnea	7.4	0.4–14.4	-	-	-	-	-	-	-	-	-	-	-	-	-	-	3.9	1.4–9.2
Ear pain	9.3	1.5–17	-	-	-	-	-	-	-	-	-	-	-	-	-	-	2	1.8–5.8
Epistaxis	7.5	4.3–10.7	3	0–6	13	4–30	-	-	-	-	5.9	2.7–9.2	-	-	-	-	4.36	2.5–8.1
Fatigue	7.4	0.4–14.4	-	-	-	-	-	-	-	-	-	-	-	-	-	-	2	1.8–5.8
Fever	3	0–5	-	-	-	-	13	2–27	-	-	-	-	-	-	-	-	5.35	1.7–10.2
Fracture	5	0–14.6	-	-	-	-	-	-	-	-	-	-	-	-	-	-	4.5	0.7–16.9
Gastroenteritis	-	-	7	6–19	-	-	-	-	-	-	-	-	-	-	-	-	5.6	1.9–20.5
General myalgia	-	-	7	6–19	-	-	-	-	-	-	-	-	-	-	-	-	5.6	1–20.5
Headache	13.8	3–24.7	13	7–33	12	0–23	7	1–14	-	-	8.4	4.6–12.2	-	-	9	8–26	8.42	5.3–13.9
Increased serum cholesterol	-	-	-	-	-	-	-	-	-	-	-	-	7	2–17	-	-	7.1	2.4–16.7
Increased serum TAG	-	-	-	-	-	-	-	-	-	-	-	-	2	1–6	-	-	1.7	0.3–6.5
Any infection	44	33.4–54.7	-	-	-	-	-	-	-	-	-	-	-	-	-	-	41.18	-
Injection site reaction	-	-	2	1–4	22	11–55	1	1–3	-	-	-	-	-	-	18	5–41	22.2	4.9–49.4
Insomnia	6	1–12	-	-	-	-	-	-	-	-	-	-	-	-	-	-	1	0.1–3.6
Jaundice	-	-	3	2–12	-	-	-	-	-	-	-	-	-	-	-	-	12.5	10.4–35.4
Laryngeal pain	-	-	-	-	-	-	-	-	-	-	-	-	2	1–6	-	-	7.1	2.4–16.7
Left ulnar hypoesthesia	-	-	7	6–19	-	-	-	-	-	-	-	-	-	-	-	-	5.6	0.9–20.5
Malignant neoplasm	-	-	1	0–2	-	-	-	-	-	-	-	-	-	-	-	-	0.4	0.4–1.1
Mild increase in thyroid hormones	5	5–15	-	-	-	-	-	-	-	-	-	-	-	-	-	-	4.5	0.7–16.9
Nasal congestion	-	-	3	0–6	-	-	-	-	-	-	-	-	-	-	27	1–54	0.6	0.02–4
Nasal obstruction	-	-	20	0–40	-	-	-	-	-	-	-	-	-	-	-	-	37.5	4–71
Nasal polyps	4	1–7	3	0–6	3	1–4	-	-	-	-	-	-	-	-	-	-	7.5	2.7–12.2
Nasopharyngitis	18	8–29	6	2–10	28	5–62	20	3–36	-	-	17.7	12.5–23	-	-	-	-	12.9	10.2–21.3
Nausea	7	0–14	-	-	-	-	-	-	-	-	-	-	-	-	-	-	3.9	1.4–9.2
Oropharyngeal pain	8	4–11	-	-	23	8–38	-	-	-	-	-	-	-	-	-	-	6.1	2.7–9.5
Otitis media	3	0–5	13	4–31	-	-	4	3–14	-	-	-	-	-	-	-	-	5.1	2.3–7.9
Pain in extremity	5	5–15	-	-	-	-	-	-	-	-	-	-	-	-	9	8–26	6.3	4.3–16.8
Pharyngitis	5	4–15	-	-	-	-	-	-	-	-	4.4	1.6–7.3	-	-	-	-	2.49	0.7–16.9
Pneumonia	-	-	-	-	-	-	4	2–14	-	-	-	-	2	1–6	-	-	7.5	7–15.6
Procedural pain	-	-	-	-	-	-	-	-	-	-	-	-	-	-	18	5–41	11.1	9.4–31.6
Red swollen eyes	2	1–9	-	-	-	-	-	-	-	-	-	-	-	-	-	-	10	8.6–28.6
Rhinitis	-	-	3	0–6	-	-	-	-	-	-	-	-	-	-	-	-	3	0.1–5.8
Rhinorrhea	6	1–12	-	-	-	-	-	-	-	-	-	-	-	-	-	-	1	0.1–3.6
Shortness of breath	5	4–15	13	4–31	-	-	-	-	-	-	-	-	-	-	-	-	6.3	4.5–17.2
Shoulder pain	-	-	7	6–19	-	-	-	-	-	-	-	-	-	-	-	-	5.6	0.9–20.5
Sinusitis	5	2–8	3	1–12	-	-	9	4–15	-	-	-	-	-	-	27	1–54	6.3	2.1–10.6
Tinnitus	-	-	-	-	-	-	-	-	-	-	-	-	7	2–17	-	-	1.7	0.3–6.5
Toothache	-	-	-	-	-	-	-	-	-	-	-	-	2	1–6	-	-	7.1	2.4–16.7
Upper GI pain	-	-	1	1–2	-	-	-	-	-	-	-	-	-	-	-	-	3	1–5.8
URTI	6	3–9	-	-	13	1–25	3	1–5	62	13–75	9.4	5.4–13.4	18	4–32	27	1–54	5.83	3.5–9.2
UTI	-	-	-	-	-	-	8	7–24	-	-	-	-	-	-	-	-	3.8	0.6–14.3

GI: Gastrointestinal; URTI: Upper Respiratory Tract Infection; UTI: Urinary Tract Infection; CI: Confidence Interval.

## Data Availability

No new data were created or analyzed in this study.

## Supplementary Materials

The following supporting information can be downloaded at: https://www.mdpi.com/article/10.3390/ph18101455/s1, Table S1: A detailed description of the search query employed in the systematic literature search of randomized trials reporting biological therapy use in patients with CRSwN. Table S2: A list of excluded articles in the full-text screening phase with description of the reasons of their exclusion. Table S3: A list of outcomes reported by RCTs comparing various biological therapies in CRSwNP not eligible for meta-analysis. Table S4: The number of patients analyzed in each outcome analysis. Figure S1: A graph showing the network of comparisons between biological therapies for CRSwNP in the literature.

## Abbreviations

The following abbreviations are used in this manuscript:

AE	Adverse event
CRSwNP	Chronic rhinosinusitis with nasal polyps
CT	Computed tomography
ESS	Endoscopic sinus surgery
LOCF	Last observation carried forward
MCID	Minimal clinically important difference
MD	Mean difference
NCS	Nasal congestion score
NMA	Network meta-analysis
NP	Nasal polyp
NPS	Nasal polyp score
OR	Odds ratio
QoL	Quality of life
RCT	Randomized controlled trial
SAE	Serious adverse event
SC	Subcutaneous
SCS	Systemic corticosteroid
SNOT-22	Sino-Nasal outcome test-22
SUCRA	Surface under the cumulative ranking curve
TNSS	Total nasal symptom score
UPSIT	University of Pennsylvania smell identification test
VAS	Visual analog scale
