# Supplementary material for: Optimizing Biologic Treatment Selection in Chronic Rhinosinusitis with Nasal Polyps: A Network Meta-Analysis of Efficacy and Safety Across 22 RCTs
Source: Pharmaceuticals (Basel). 2025 Sep 28;18(10):1455. doi: 10.3390/ph18101455 (PMC12566755; doi:10.3390/ph18101455)
Supplement: Supplementary file 1 [file pharmaceuticals-18-01455-s001.zip › pharmaceuticals-3803666-supplementary.pdf]

Supplementary File

Table S1. A detailed description of the search query employed in the systematic literature search of randomized trials reporting biological therapy use in patients with CRSwNP

Database	No.	Search Query	Results
**PubMed [Date of search: 05/19/2024]**			
	#1	rhinosinusitis[tiab] OR rhino-sinusitis[tiab] OR sinusitis[tiab] OR "Rhinosinusitis"[Mesh] OR "Sinusitis"[Mesh]	35728
	#2	“nasal polyp*”[tiab] OR "Nasal Polyps"[Mesh]	11542
	#3	#1 AND #2	6664
	#4	CRSwNP[tiab]	2156
	#5	#3 OR #4	6734
	#6	Dupilumab[tiab] OR SAR231893[tiab] OR SAR-231893[tiab] OR Dupixent[tiab] OR REGN668[tiab] OR REGN-668[tiab] OR Mepolizumab[tiab] OR Bosatria[tiab] OR SB-240563[tiab] OR SB240563[tiab] OR Nucala[tiab] OR Omalizumab[tiab] OR Xolair[tiab] OR Reslizumab[tiab] OR SCH-55700[tiab] OR SCH55700[tiab] OR "SCH 55700"[tiab] OR CEP-38072[tiab] OR CEP38072[tiab] OR Cinqair[tiab] OR DCP-835[tiab] OR DCP835[tiab] OR "DCP 835"[tiab] OR Benralizumab[tiab] OR MEDI-563[tiab] OR "MEDI 563"[tiab] OR Fasenra[tiab] OR BIW-8405[tiab] OR Tezepelumab[tiab] OR tezepelumab-ekko[tiab] OR tezspire[tiab] OR MEDI-9929[tiab] OR MEDI-19929[tiab] OR MEDI9929[tiab] OR AMG-157[tiab] OR Etokimab[tiab] OR Lebrikizumab[tiab] OR RO-5490255[tiab] OR RG-3637[tiab] OR TNX-650[tiab] OR MILR1444A[tiab] OR MILR-1444A[tiab] OR PRO301444[tiab] OR PRO-301444[tiab] OR Tralokinumab[tiab] OR CAT-354[tiab] OR AK001[tiab] OR anti-Siglec-8[tiab] OR Astegolimab[tiab] OR MSTT1041A[tiab] OR MSTT-1041A[tiab] OR RO-7187807[tiab] OR RO7187807[tiab] OR RG6149[tiab] OR RG-6149[tiab] OR “anti-immunoglobulin E”[tiab] OR anti-interleukin-4[tiab] OR anti-interleukin-13[tiab] OR “anti-interleukin-4 receptor”[tiab] OR anti-interleukin-5[tiab] OR “anti-interleukin-5 receptor”[tiab] OR “anti-interleukin-13”[tiab] OR “anti-interleukin-33”[tiab] OR “anti-thymic stromal lymphopoietin”[tiab] OR “Anti-IgE”[tiab] OR “anti-IL5”[tiab] OR “anti-IL4/IL13”[tiab] OR ASA-D[tiab] OR "dupilumab" [Supplementary Concept] OR "mepolizumab" [Supplementary Concept] OR "Omalizumab"[Mesh] OR "reslizumab" [Supplementary Concept] OR "benralizumab" [Supplementary Concept] OR "tezepelumab" [Supplementary Concept] OR "lebrikizumab" [Supplementary Concept] OR "tralokinumab" [Supplementary Concept] OR "astegolimab" [Supplementary Concept]	11473
	#7	Random*[tiab] OR "Randomized Controlled Trial" [Publication Type]	1650845
	#8	#5 AND #6 AND #7	97
**Scopus [Date of search: 05/19/2024]**			

#1	TITLE-ABS-KEY (rhinosinusitis) OR TITLE-ABS-KEY (rhino-sinusitis) OR TITLE-ABS-KEY (sinusitis)	59989
#2	TITLE-ABS-KEY (“nasal polyp*”)	12742
#3	#1 AND #2	7534
#4	TITLE-ABS-KEY (CRSwNP)	813
#5	#3 OR #4	7569
#6	TITLE-ABS-KEY (Dupilumab) OR TITLE-ABS-KEY (SAR231893) OR TITLE-ABS-KEY (SAR-231893) OR TITLE-ABS-KEY (Dupixent) OR TITLE-ABS-KEY (REGN668) OR TITLE-ABS-KEY (REGN-668) OR TITLE-ABS-KEY (Mepolizumab) OR TITLE-ABS-KEY (Bosatria) OR TITLE-ABS-KEY (SB-240563) OR TITLE-ABS-KEY (SB240563) OR TITLE-ABS-KEY (Nucala) OR TITLE-ABS-KEY (Omalizumab) OR TITLE-ABS-KEY (Xolair) OR TITLE-ABS-KEY (Reslizumab) OR TITLE-ABS-KEY (SCH-55700) OR TITLE-ABS-KEY (SCH55700) OR TITLE-ABS-KEY ("SCH 55700") OR TITLE-ABS-KEY (CEP-38072) OR TITLE-ABS-KEY (CEP38072) OR TITLE-ABS-KEY (Cinqair) OR TITLE-ABS-KEY (DCP-835) OR TITLE-ABS-KEY (DCP835) OR TITLE-ABS-KEY ("DCP 835") OR TITLE-ABS-KEY (Benralizumab) OR TITLE-ABS-KEY (MEDI-563) OR TITLE-ABS-KEY ("MEDI 563") OR TITLE-ABS-KEY (Fasenra) OR TITLE-ABS-KEY (BIW-8405) OR TITLE-ABS-KEY (Tezepelumab) OR TITLE-ABS-KEY (tezepelumab-ekko) OR TITLE-ABS-KEY (tezspire) OR TITLE-ABS-KEY (MEDI-9929) OR TITLE-ABS-KEY (MEDI-19929) OR TITLE-ABS-KEY (MEDI9929) OR TITLE-ABS-KEY (AMG-157) OR TITLE-ABS-KEY (Etokimab) OR TITLE-ABS-KEY (Lebrikizumab) OR TITLE-ABS-KEY (RO-5490255) OR TITLE-ABS-KEY (RG-3637) OR TITLE-ABS-KEY (TNX-650) OR TITLE-ABS-KEY (MILR1444A) OR TITLE-ABS-KEY (MILR-1444A) OR TITLE-ABS-KEY (PRO301444) OR TITLE-ABS-KEY (PRO-301444) OR TITLE-ABS-KEY (Tralokinumab) OR TITLE-ABS-KEY (CAT-354) OR TITLE-ABS-KEY (AK001) OR TITLE-ABS-KEY (anti-Siglec-8) OR TITLE-ABS-KEY (Astegolimab) OR TITLE-ABS-KEY (MSTT1041A) OR TITLE-ABS-KEY (MSTT-1041A) OR TITLE-ABS-KEY (RO-7187807) OR TITLE-ABS-KEY (RO7187807) OR TITLE-ABS-KEY (RG6149) OR TITLE-ABS-KEY (RG-6149) OR TITLE-ABS-KEY (“anti-immunoglobulin E”) OR TITLE-ABS-KEY (anti-interleukin-4) OR TITLE-ABS-KEY (anti-interleukin-13) OR TITLE-ABS-KEY (“anti-interleukin-4 receptor”) OR TITLE-ABS-KEY (anti-interleukin-5) OR TITLE-ABS-KEY (“anti-interleukin-5 receptor”) OR TITLE-ABS-KEY (“anti-interleukin-13”) OR TITLE-ABS-KEY (“anti-interleukin-33”) OR TITLE-ABS-KEY (“anti-thymic stromal lymphopoietin”) OR TITLE-ABS-KEY (“Anti-IgE”) OR TITLE-ABS-KEY (“anti-IL5”) OR TITLE-ABS-KEY (“anti-IL4/IL13”) OR TITLE-ABS-KEY (ASA-D)	20012
#7	TITLE-ABS-KEY (Random*)	3322770
#8	#5 AND #6 AND #7	163
**Web of Science [Date of search: 05/19/2024]**		
#1	AB=rhinosinusitis OR AB=rhino-sinusitis OR AB=sinusitis	20553

#2	AB="nasal polyp*"	6768
#3	#1 AND #2	4263
#4	AB=CRSwNP	1897
#5	#3 OR #4	4406
#6	AB=Dupilumab OR AB=SAR231893 OR AB=SAR-231893 OR AB=Dupixent OR AB=REGN668 OR AB=REGN-668 OR AB=Mepolizumab OR AB=Bosatria OR AB=SB-240563 OR AB=SB240563 OR AB=Nucala OR AB=Omalizumab OR AB=Xolair OR AB=Reslizumab OR AB=SCH-55700 OR AB=SCH55700 OR AB="SCH 55700" OR AB=CEP-38072 OR AB=CEP38072 OR AB=Cinqair OR AB=DCP-835 OR AB=DCP835 OR AB="DCP 835" OR AB=Benralizumab OR AB=MEDI-563 OR AB="MEDI 563" OR AB=Fasenra OR AB=BIW-8405 OR AB=Tezepelumab OR AB=tezepelumab-ekko OR AB=tezspire OR AB=MEDI-9929 OR AB=MEDI-19929 OR AB=MEDI9929 OR AB=AMG-157 OR AB=Etokimab OR AB=Lebrikizumab OR AB=RO-5490255 OR AB=RG-3637 OR AB=TNX-650 OR AB=MILR1444A OR AB=MILR-1444A OR AB=PRO301444 OR AB=PRO-301444 OR AB=Tralokinumab OR AB=CAT-354 OR AB=AK001 OR AB=anti-Siglec-8 OR AB=Astegolimab OR AB=MSTT1041A OR AB=MSTT-1041A OR AB=RO-7187807 OR AB=RO7187807 OR AB=RG6149 OR AB=RG-6149 OR AB="anti-immunoglobulin E" OR AB=anti-interleukin-4 OR AB=anti-interleukin-13 OR AB="anti-interleukin-4 receptor" OR AB=anti-interleukin-5 OR AB="anti-interleukin-5 receptor" OR AB="anti-interleukin-13" OR AB="anti-interleukin-33" OR AB="anti-thymic stromal lymphopoietin" OR AB="Anti-IgE" OR AB="anti-IL5" OR AB="anti-IL4/IL13" OR AB=ASA-D	7947
#7	AB=Random*	2187348
#8	#5 AND #6 AND #7	72
**CENTRAL Registry [Date of search: 05/19/2024]**		
#1	rhinosinusitis OR rhino-sinusitis OR sinusitis	4770
#2	"nasal polyp" OR "nasal polyps" OR "nasal polyposis" OR polyp OR polyps	8116
#3	#1 AND #2	1100
#4	CRSwNP	369
#5	#3 OR #4	1112
#6	Dupilumab OR SAR231893 OR SAR-231893 OR Dupixent OR REGN668 OR REGN-668 OR Mepolizumab OR Bosatria OR SB-240563 OR SB240563 OR Nucala OR Omalizumab OR Xolair OR Reslizumab OR SCH-55700 OR SCH55700 OR "SCH 55700" OR CEP-38072 OR CEP38072 OR Cinqair OR DCP-835 OR DCP835 OR "DCP 835" OR Benralizumab OR MEDI-563 OR "MEDI 563" OR Fasenra OR BIW-8405 OR Tezepelumab OR tezepelumab-ekko OR tezspire OR MEDI-9929 OR MEDI-19929 OR MEDI9929 OR AMG-157 OR Etokimab OR Lebrikizumab OR RO-5490255 OR RG-3637 OR TNX-650 OR MILR1444A OR MILR-1444A OR PRO301444 OR PRO-301444 OR Tralokinumab OR CAT-354 OR AK001 OR anti-Siglec-8 OR Astegolimab OR MSTT1041A OR MSTT-1041A OR RO-7187807 OR RO7187807 OR RG6149 OR RG-6149 OR "anti-	3661

		immunoglobulin E” OR anti-interleukin-4 OR anti-interleukin-13 OR “anti-interleukin-4 receptor” OR anti-interleukin-5 OR “anti-interleukin-5 receptor” OR “anti-interleukin-13” OR “anti-interleukin-33” OR “anti-thymic stromal lymphopoietin” OR “Anti-IgE” OR “anti-IL5” OR “anti-IL4/IL13” OR ASA-D	
	#7	Random*	1422676
	#8	#5 AND #6 AND #7	194
**Clinicaltrials.gov [Date of search: 05/19/2024]**			
	Condition/disease	Chronic Rhinosinusitis With Nasal Polyps	-
	Other terms	-	-
	Intervention/treatment	Dupilumab OR SAR231893 OR SAR-231893 OR Dupixent OR REGN668 OR REGN-668 OR Mepolizumab OR Bosatria OR SB-240563 OR SB240563 OR Nucala OR Omalizumab OR Xolair OR Reslizumab OR SCH-55700 OR SCH55700 OR "SCH 55700" OR CEP-38072 OR CEP38072 OR Cinqair OR DCP-835 OR DCP835 OR "DCP 835" OR Benralizumab OR MEDI-563 OR "MEDI 563" OR Fasenra OR BIW-8405 OR Tezepelumab OR tezepelumab-ekko OR tezspire OR MEDI-9929 OR MEDI-19929 OR MEDI9929 OR AMG-157 OR Etokimab OR Lebrikizumab OR RO-5490255 OR RG-3637 OR TNX-650 OR MILR1444A OR MILR-1444A OR PRO301444 OR PRO-301444 OR Tralokinumab OR CAT-354 OR AK001 OR anti-Siglec-8 OR Astegolimab OR MSTT1041A OR MSTT-1041A OR RO-7187807 OR RO7187807 OR RG6149 OR RG-6149 OR “anti-immunoglobulin E” OR anti-interleukin-4 OR anti-interleukin-13 OR “anti-interleukin-4 receptor” OR anti-interleukin-5 OR “anti-interleukin-5 receptor” OR “anti-interleukin-13” OR “anti-interleukin-33” OR “anti-thymic stromal lymphopoietin” OR “Anti-IgE” OR “anti-IL5” OR “anti-IL4/IL13” OR ASA-D	-
	Total	Filter applied: Completed	16
**Google Scholar [Date of search: 05/19/2024]**			
	With all of the words	Random rhinosinusitis with nasal polyp	-
	With the exact phrase	-	-
	With at least one of the words	Dupilumab OR SAR231893 OR SAR-231893 OR Dupixent OR REGN668 OR REGN-668 OR Mepolizumab OR Bosatria OR SB-240563 OR SB240563 OR Nucala OR Omalizumab OR Xolair OR Reslizumab OR SCH-55700 OR SCH55700 OR "SCH 55700" OR CEP-38072 OR CEP38072 OR Cinqair OR DCP-835 OR DCP835 OR "DCP 835" OR Benralizumab OR MEDI-563 OR "MEDI 563" OR Fasenra OR BIW-8405 OR Tezepelumab OR tezepelumab-ekko OR tezspire OR MEDI-9929 OR MEDI-19929 OR MEDI9929 OR AMG-157 OR Etokimab OR Lebrikizumab OR RO-5490255 OR RG-3637 OR TNX-650 OR MILR1444A OR MILR-1444A OR PRO301444 OR PRO-301444 OR Tralokinumab OR CAT-354 OR AK001 OR anti-Siglec-8 OR Astegolimab OR MSTT1041A OR MSTT-1041A OR RO-7187807 OR RO7187807 OR RG6149 OR RG-6149 OR “anti-immunoglobulin E” OR anti-interleukin-4 OR anti-interleukin-13 OR “anti-interleukin-4 receptor” OR anti-interleukin-5 OR “anti-interleukin-5 receptor” OR “anti-interleukin-13” OR “anti-interleukin-33”	-

	OR “anti-thymic stromal lymphopoietin” OR “Anti-IgE” OR “anti-IL5” OR “anti-IL4/IL13” OR ASA-D	
Total	As per recent recommendations, only the first 200 records were selected	200

CENTRAL: Cochrane Central Register of Controlled Trials; CRSwNP: Chronic Rhinosinusitis with Nasal Polyps.

Table S2. A list of excluded articles in the full-text screening phase with description of the reasons of their exclusion

ID	Author	YOP	Title	Journal	DOI	Decision
**28**	C. H. Bachert, P. Mullol, J. Hamilos, D. Naclerio, R. Joish, V. N. Mannent, L. Evans, R. E. Grabher, A. Abbe, A. et al.,	2016	Dupilumab improves patient-reported outcomes in chronic sinusitis with nasal polyps patients with comorbid asthma: results from a phase 2a trial	European respiratory journal	10.1183/13993003.congress-2016.OA251	Abstract-only publication
**240**	P. B. Gevaert, Claus Maspero, Jorge Fernando Cuevas, Mandy Steele, David Acharya, Sandip Altman, Pablo	2022	Phase 3b randomized controlled trial of fevipiprant in patients with nasal polyposis with asthma (THUNDER)	Journal of Allergy and Clinical Immunology		Abstract-only publication
**291**	C. B. Hopkins, K. M. Heffler, E. Cohen, N. A. Olze, H. Khan, A. H. Msihid, J. Siddiqui, S. Nash, S. Jacob-Nara, J. A. et al.,	2022	Improvement in Health-Related Quality of Life with Dupilumab in Patients with Moderate-to-Severe Asthma with Comorbid Chronic Rhinosinusitis with/without Nasal Polyps: an Analysis of the QUEST Study	Journal of asthma and allergy	10.2147/JAA.S363527	Abstract-only publication
**292**	C. B. Hopkins, K. Heffler, E. Cohen, N. Olze, H. Khan, A. Msihid, J. Siddiqui, S. Nash, S. Jacob-Nara, J. A. et al.,	2022	Dupilumab Improved Health-Related Quality of Life in Asthma Patients with Comorbid Chronic Rhinosinusitis: QUEST Study	American journal of respiratory and critical care medicine	10.1164/ajrccm-conference.2022.205.1_MeetingAbstracts.A4845	Abstract-only publication
**23**	C. D. Bachert, M. Mullol, J. Hellings, P. W. Cervin, A. Sher, L. Bosso, J. V. Lee, S. E. Maspero, J. F. Fujieda, S. et al.,	2019	A Randomized Phase 3 Study, Sinus-52, Evaluating the Efficacy and Safety of Dupilumab in Patients with Severe Chronic Rhinosinusitis with Nasal Polyps	Journal of allergy and clinical immunology	10.1016/j.jaci.2018.12.980	Abstract-only publication
**270**	J. K. B. Han, C. Desrosiers, M. Laidlaw, T. M. Hopkins, C. Fokkens, W. J. Paggiaro, P. Cho, S. H. Olze, H. Greos, L. S. et al.,	2019	Efficacy and safety of dupilumab in patients with chronic rhinosinusitis with nasal polyps: results from the randomized phase 3 sinus-24 study	Journal of allergy and clinical immunology	10.1016/j.jaci.2018.12.948	Abstract-only publication
**307**	P. M. Howarth, J. Bachert, C. Fokkens, W. Smith, S. Keeley, T. Zhang, L. Chan, R.	2024	Impact of Mepolizumab on Sleep Disturbance in Patients With Chronic Rhinosinusitis With Nasal Polyps and/or Asthma: analysis From SYNAPSE and MUSCA	Journal of allergy and clinical immunology	10.1016/j.jaci.2023.11.687	Abstract-only publication
**14**	E. A. Atayik, G.	2022	A Single Center Experience of Super-Responders Among Severe Asthma Patients Receiving Treatment with Mepolizumab	TURKISH THORACIC JOURNAL	10.5152/TurkThoracJ.2022.22023	Asthma patients without CRSwNP (or no subgroup analysis based on CRSwNP status)
**118**	G. W. H. Canonica, T. W. Chanez, P. Menzella, F. Louis, R. Cosio, B. G. Lugogo, N. L. Mohan, A. Burden, A. McDermott, L. et al.,	2020	Benralizumab efficacy for severe, eosinophilic asthma with a diagnosis of nasal polyposis: results from the phase IIIb ANDHI trial	Allergy	10.1111/all.14505	Asthma patients without CRSwNP (or no subgroup analysis based on CRSwNP status)
**254**	P. G. P. Gibson, C. M. Chupp, G. L. Bradford, E. S. Forshag, M. Mallett, S. A. Yancey, S. W. Smith, S. G. Bel, E. H.	2021	Mepolizumab improves clinical outcomes in patients with severe asthma and comorbid conditions	Respiratory research	10.1186/s12931-021-01746-4	Asthma patients without CRSwNP (or no subgroup analysis based on CRSwNP status)
**462**	H. L. Ortega, H. Suruki, R. Albers, F. Gordon, D. Yancey, S.	2014	Cluster analysis and characterization of response to mepolizumab. A step closer to personalized medicine for patients with severe asthma	Ann Am Thorac Soc	10.1513/AnnalsATS.201312-454OC	Asthma patients without CRSwNP (or no subgroup

						analysis based on CRSwNP status)
**250**	P. S. Gevaert, R. Corren, J. Han, J. Mullol, J. Lee, S. Zhao, R. Howard, M. Wong, K. Islam, L. et al.,	2020	CONTINUED SAFETY/EFFICACY OF OMALIZUMAB IN CHRONIC RHINOSINUSITIS WITH NASAL POLYPS: AN OPEN-LABEL EXTENSION STUDY	Annals of allergy, asthma and immunology	10.1016/j.anai.2020.08.066	Conference presentation
**19**	C. C. Bachert, S. Laidlaw, T. Swanson, B. Harel, S. Mannent, L. Amin, N. Jagerschmidt, A.	2020	Dupilumab Reduces Blood, Urine, and Nasal Biomarkers of Type 2 Inflammation in Patients With Chronic Rhinosinusitis With Nasal Polyps in the Phase 3 SINUS-52 Trial	Journal of allergy and clinical immunology	10.1016/j.jaci.2019.12.324	Conference presentation
**20**	C. C. Bachert, J. Lee, S. E. Zhang, H. Harel, S. Cunoosamy, D. Khan, A. H. Jacob-Nara, J. A. Siddiqui, S. Nash, S.	2021	Association between dupilumab effect on nasal polyp score and biomarkers of type 2 inflammation in patients with chronic rhinosinusitis with nasal polyps in the phase 3 sinus-24 and sinus-52 trials	American journal of respiratory and critical care medicine	10.1164/ajrccm-conference.2021.203.1_MeetingAbstracts.A1343	Conference presentation
**25**	C. F. Bachert, J. W. Jankowski, R. Cervin, U. A. Laidlaw, M. T. Lee, E. S. Zhang, M. Lu, X. Amin, N. Patel, N. et al.,	2019	Dupilumab efficacy in patients with severe chronic rhinosinusitis with nasal polyposis: pooled results from the SINUS-24 and SINUS-52 phase 3 studies	Allergy	10.1111/all.13957	Conference presentation
**39**	C. H. Bachert, J. K. Desrosiers, M. Hellings, P. W. Mao, X. Zhang, M. Amin, N. Mannent, L. P. Kamat, S. Khan, A. H.	2020	Dupilumab improves asthma control as assessed by total and individual item scores of the 6-item asthma control questionnaire in patients with severe chronic rhinosinusitis with nasal polyps and comorbid asthma: pooled results from the sinus-24 and sinus-52 phase 3 studies	American journal of respiratory and critical care medicine		Conference presentation
**44**	C. H. Bachert, J. K. Desrosiers, M. Gevaert, P. Heffler, E. Hopkins, C. Tversky, J. R. Barker, P. Cohen, D. Emson, C. et al.,	2021	Efficacy and safety of benralizumab for the treatment of chronic rhinosinusitis with nasal polyps: results from the phase III OSTRO trial	Allergy	10.1111/all.15093	Conference presentation
**47**	C. H. Bachert, C. Han, J. Fokkens, W. Mannent, L. Khan, A. Msihid, J. Mujumdar, U. Kamat, S. Nash, S. et al.,	2022	Symptom Free Days In Patients With Severe Chronic Rhinosinusitis with Nasal Polyps Treated with Dupilumab	Journal of allergy and clinical immunology	10.1016/j.jaci.2021.12.484	Conference presentation
**52**	C. H. Bachert, C. Han, J. Fokkens, W. Mannent, L. Khan, A. Msihid, J. Nair, R. Kamat, S. Nash, S. et al.,	2023	Symptom-free days in patients with severe chronic rhinosinusitis with nasal polyps treated with dupilumab	Allergy	10.1111/all.15616	Conference presentation
**66**	C. S. Bachert, A. Han, J. Schlosser, R. Sowerby, L. Hopkins, C. Maspero, J. Kante, O. Karidi-Andrioti, D. Chaker, A.	2020	Mepolizumab for chronic rhinosinusitis with nasal polyps: comorbid asthma, nsaid exacerbated respiratory disease, eosinophil stratification	Annals of allergy, asthma and immunology	10.1016/j.anai.2020.08.161	Conference presentation
**99**	L. S. Borish, J. Baylis, L. Howarth, P. Chan, R. Dsilva, P. Hopkins, C. Han, J.	2024	Profiling Symptomatic and Quality of Life Benefits of Mepolizumab for Patients With Chronic Rhinosinusitis With Nasal Polyps (CRSwNP) in the SYNAPSE Trial	Journal of allergy and clinical immunology	10.1016/j.jaci.2023.11.807	Conference presentation
**157**	J. W. Corren, P. H. Gevaert, P. Bachert, C. Mullol, J. Jaumont, X. Zhao, R. Saenz, R. Islam, L. Omachi, T.	2020	Omalizumab responders analysis in nasal polyposis: a supplementary analysis of the co-primary endpoints of POLYP 1 and POLYP 2 trials	Allergy	10.1111/all.14508	Conference presentation
**165**	C. Y. Damask, B. Zhao, R. Saenz, R. Millette, L. Rajput, Y. Franzese, C.	2020	IMPACT OF OMALIZUMAB ON QUALITY OF LIFE IN PATIENTS WITH CHRONIC RHINOSINUSITIS WITH NASAL POLYPS	Annals of allergy, asthma and immunology	10.1016/j.anai.2020.08.166	Conference presentation
**172**	L. K. de Prado Gomez, A. Peters, A. Bachert, C. Wagenmann, M. Heffler, E. Hopkins, C. Hellings, P. Zhang, M. Xing, J. et al.,	2022	EValuating trEatment RESponses of dupilumab versus omalizumab in Type 2 patients: the EVEREST Trial	Journal of allergy and clinical immunology	10.1016/j.jaci.2021.12.191	Conference presentation
**181**	M. B. Desrosiers, C. Hellings, P. W. Hopkins, C. Olze, H. Han, J. K. Lee, S. E. Zhang, M. Lu, X. Amin, N. et al.,	2019	Dupilumab reduces need for systemic corticosteroid use, sinonasal surgery in patients with severe chronic rhinosinusitis with nasal polyps: pooled results from the SINUS-24, SINUS-52 phase 3 studies	Allergy	10.1111/all.13959	Conference presentation
**182**	M. B. Desrosiers, C. Hellings, P. Hopkins, C. Olze, H. Han, J. K. Lee, S. E. Zhang, M. Lu, X. Amin, N. et al.,	2020	Dupilumab reduces systemic corticosteroid use and sinonasal surgery in patients with severe chronic rhinosinusitis with nasal polyps: pooled results from the phase 3 SINUS-24 and SINUS-52 studies	Allergy, asthma and clinical immunology	10.1186/s13223-020-00445-x	Conference presentation

**208**	H. U. Euctr	2021	EVEREST: eValuating trEatment RESponses of dupilumab versus omalizumab in Type 2 patients	https://trialsearch.who.int/Trial2.aspx?TrialID=EUCTR2021-000829-27-HU		Conference presentation
**238**	P. B. Gevaert, C. Desrosiers, M. Mullol, J. Maspero, J. Zhang, M. Mao, X. Kamat, S. Khan, A. Amin, N. et al.,	2019	DUPILUMAB IMPROVES PATIENT-REPORTED OUTCOMES IN CHRONIC RHINOSINUSITIS WITH NASAL POLYPS AND COMORBID ASTHMA: SINUS-24/SINUS-52 TRIALS	Annals of allergy, asthma and immunology	10.1016/j.anai.2019.08.343	Conference presentation
**239**	P. B. Gevaert, C. Desrosiers, M. Mullol, J. Maspero, J. F. Zhang, M. Mao, X. Kamat, S. Khan, A. H. Amin, N. et al.,	2020	Dupilumab improves patient-reported outcomes in chronic rhinosinusitis with nasal polyps with and without comorbid asthma: SINUS-24/SINUS-52 Trials	Canadian journal of respiratory, critical care, and sleep medicine	10.1080/24745332.2020.1750227	Conference presentation
**244**	P. C. Gevaert, T. Holweg, C. Ko, J. Montalto, K. Olsson, P. Millette, L. Blaiss, M.	2021	OMALIZUMAB IMPROVED SYMPTOMS IN PATIENTS WITH NASAL POLYPS WITH AND WITHOUT PHYSICIAN-REPORTED ALLERGIC COMORBIDITIES	Annals of allergy, asthma and immunology	10.1016/j.anai.2021.08.160	Conference presentation
**258**	S. N. R.-G. Gonzalez-Diaz, L.	2014	Omalizumab efficiency in patients with allergic rhinitis and chronic sinusitis	World Allergy Organization journal		Conference presentation
**279**	T. W. C. Harrison, P. Menzella, F. Canonica, G. W. Louis, R. Cosio, B. G. Lugogo, N. L. Mohan, A. Burden, A. McDermott, L. et al.,	2020	Exacerbation reduction and early and sustained improvements in SGRQ, lung function, and symptoms of nasal polyposis with benralizumab for severe, eosinophilic asthma: phase IIIb andhi trial	American journal of respiratory and critical care medicine		Conference presentation
**311**	M. L. Humbert, M. C. Moore, W. C. Bel, E. H. Kaneko, N. Smith, S. G. Martin, N. Gilson, M. J. Mavropoulou, E. Price, R. G. et al.,	2021	Previous exacerbations predict the risk of future exacerbations after stopping versus continuing mepolizumab treatment: secondary analysis of the comet trial	American journal of respiratory and critical care medicine	10.1164/ajrccm-conference.2021.203.1_MeetingAbstracts.A1445	Conference presentation
**316**	J. H. Jacobs, F. Spahn, J. Ambrose, C. Martin, N. Vong, S. Caveney, S. Cook, B. Colice, G.	2023	Tezepelumab Efficacy By SNOT-22 Score In Patients With Severe, Uncontrolled Asthma And Comorbid Nasal Polyps In NAVIGATOR	Journal of allergy and clinical immunology	10.1016/j.jaci.2022.12.058	Conference presentation
**337**	S. L. Kilty, A. Santucci, S. Yang, W. H.	2017	Prospective evaluation of omalizumab treatment for patient symptom control of chronic rhinosinusitis	Journal of allergy and clinical immunology		Conference presentation
**361**	T. M. M. Laidlaw, J. Canonica, G. W. Bachert, C. Han, J. K. Lu, X. Patel, N. Graham, N. M. H. Staudinger, H. Mannent, L. P. et al.,	2020	Dupilumab improves upper and lower airway outcomes in chronic rhinosinusitis with nasal polyps (CRSwNP) with nonsteroidal anti-inflammatory drug-exacerbated respiratory disease (NSAID-ERD): pooled results from SINUS-24, SINUS-52 phase 3 trials	Canadian journal of respiratory, critical care, and sleep medicine	10.1080/24745332.2020.1750227	Conference presentation
**363**	T. M. J. Lailaw, M. Canonica, W. Bachert, C. Han, J. K. Zhang, M. Lu, X. Patel, N. Graham, N. M. H. Staudinger, H. et al.,	2020	Dupilumab Improves Upper and Lower Airway Outcome Measures in Patients With CRSwNP With NSAID-ERD: pooled Results from the SINUS-24 and SINUS-52 Phase 3 Trials	World Allergy Organization journal	10.1016/j.waojou.2020.100398	Conference presentation
**370**	S. E. Y. Lee, B. Saenz, R. Braid, J. Millette, L. A. Rajput, Y.	2020	PRS2 PROPORTION OF PATIENTS WITH NASAL POLYPOSIS ACHIEVING CLINICALLY IMPORTANT IMPROVEMENTS IN QUALITY OF LIFE WITH OMALIZUMAB TREATMENT	Value in health	10.1016/j.jval.2020.04.1329	Conference presentation
**371**	S. T. Lee, M. Trigg, A. Han, J. Fokkens, W. Naclerio, R. Gevaert, P. Sousa, A. Howarth, P. Mayer, B. et al.,	2021	Mepolizumab Improves Health Related Quality of Life for Patients with Chronic Rhinosinusitis with Nasal Polyps: data from the SYNAPSE study	Journal of allergy and clinical immunology	10.1016/j.jaci.2020.12.458	Conference presentation
**387**	A. L. Luong, J. Klimek, L. Harvey, R. Silver, J. Smith, S. Fuller, A. Chan, R. Hellings, P.	2023	Change in nasal polyp size as an indicator of treatment response: SYNAPSE trial analysis	Journal of allergy and clinical immunology	10.1016/j.jaci.2022.12.343	Conference presentation
**392**	J. F. B. Maspero, C. Canonica, G. W. Swanson, B. N. Cho, S. H. Harel, S. Jagerschmidt, A. Zhang, M. Mao, X. Mannent, L. P. et al.,	2021	Efficacy of dupilumab in chronic rhinosinusitis with nasal polyps and comorbid asthma by baseline biomarkers of type 2 inflammation: SINUS-24 and SINUS-52 phase 3 trials	Clinical and experimental allergy	10.1111/cea.13812	Conference presentation
**393**	J. F. B. Maspero, C. Walter Canonica, G. Swanson, B. N. Cho, S. H. Harel, S. Jagerschmidt, A. Zhang, M. Mao, X. Mannent, L. P. et al.,	2021	Efficacy of dupilumab in chronic rhinosinusitis with nasal polyps and comorbid asthma by baseline biomarkers of type 2 inflammation from the pooled population of the SINUS-24 and SINUS-52 phase 3 trials	Allergy, asthma and clinical immunology	10.1186/s13223-021-00519-4	Conference presentation

**395**	J. F. K. Maspero, C. Jayawardena, S. Rowe, P. Maroni, J. Pirozzi, G. Amin, N. Graham, N. M. H. Mannent, L. Teper, A.	2017	Dupilumab efficacy in uncontrolled persistent asthma patients with history of comorbid chronic rhinosinusitis with or without nasal polyps	American journal of respiratory and critical care medicine	10.1164/ajrccm-conference.2017.B101	Conference presentation
**411**	J. B. Mullol, C. Desrosiers, M. Han, J. K. Jankowski, R. Daizadeh, N. Amin, N. Mannent, L. P. Khan, A. H. Kamat, S.	2020	Dupilumab rapidly improves sense of smell in patients with severe chronic rhinosinusitis with nasal polyps: pooled results from the SINUS-24 and SINUS-52 phase 3 studies	Allergy	10.1111/all.14506	Conference presentation
**416**	J. H. Mullol, J. Bosso, J. Mannent, L. Amin, N. Cho, S. Bachert, C.	2020	Dupilumab Treatment Improves Sense of Smell in Patients With Chronic Rhinosinusitis With Nasal Polyps – Pooled Results From the SINUS-24 and SINUS-52 Phase 3 Trials	Journal of allergy and clinical immunology	10.1016/j.jaci.2019.12.349	Conference presentation
**418**	J. L. Mullol, V. Wagenmann, M. Han, J. Sousa, A. Smith, S. Mayer, B. Yancey, S. Chan, R. Fokkens, W.	2022	Mepolizumab Induced Loss of Smell Improvement in Patients With Chronic Rhinosinusitis With Nasal Polyps From the SYNAPSE Study	Journal of allergy and clinical immunology	10.1016/j.jaci.2021.12.533	Conference presentation
**457**	S. Nsouli	2019	DUPILUMAB A HUMAN ANTI-INTERLEUKIN-4 RECEPTOR ALPHA MONOCLONAL ANTIBODY FOR UNCONTROLLED CHRONIC RHINOSINUSITIS WITH NASAL POLYPS	Annals of allergy, asthma and immunology	10.1016/j.anai.2019.08.344	Conference presentation
**458**	S. Nsouli	2020	RESLIZUMAB AN INTERLEUKIN-5 ANTAGONIST MONOCLONAL ANTIBODY FOR UNCONTROLLED CHRONIC RHINOSINUSITIS WITH NASAL POLYPS	Annals of allergy, asthma and immunology	10.1016/j.anai.2020.08.167	Conference presentation
**471**	A. H. Peters, J. Hellings, P. Heffler, E. Gevaert, P. Bachert, C. Xu, Y. Chuang, C. Mannent, L. Guyot, P. et al.,	2020	INDIRECT TREATMENT COMPARISON OF BIOLOGICS USED FOR THE TREATMENT OF CHRONIC RHINOSINUSITIS WITH NASAL POLYPS	Annals of allergy, asthma and immunology	10.1016/j.anai.2020.08.164	Conference presentation
**475**	A. T. S. Peters, Z. M. Kern, R. C. Heffler, E. Maspero, J. F. Crampette, L. Fujieda, S. Lane, A. P. Zhang, H. Nash, S. et al.,	2021	Improvement in patient-reported 'taste' and association with smell in dupilumab-treated severe chronic rhinosinusitis with nasal polyps patients from the SINUS-24 and SINUS-52 trials	Allergy	10.1111/all.15095	Conference presentation
**479**	A. W. Peters, M. Bernstein, J. A. Zhang, H. Khan, A. H. Nash, S. Jacob-Nara, J. A. Siddiqui, S.	2021	Efficacy of dupilumab in patients with chronic rhinosinusitis with nasal polyps and allergic rhinitis	American journal of respiratory and critical care medicine	10.1164/ajrccm-conference.2021.203.1_MeetingAbstracts.A1340	Conference presentation
**516**	S. P. Smith, R. G. Mollo, M. R. Howarth, P. Yancey, S. W. Cavaliere, C.	2022	Clinical Burden of Chronic Rhinosinusitis with Nasal Polyps in Patients with Severe Eosinophilic Asthma: a Post Hoc Analysis of Data from MENSA and COSMOS	American journal of respiratory and critical care medicine	10.1164/ajrccm-conference.2022.205.1_MeetingAbstracts.A4829	Conference presentation
**517**	Z. L. Soler, A. Patel, Z. Mattos, J. Xia, C. Khan, A. Nash, S.	2022	ASSOCIATION BETWEEN SMELL LOSS, DISEASE BURDEN, AND DUPILUMAB EFFICACY IN CHRONIC RHINOSINUSITIS WITH NASAL POLYPS	Annals of allergy, asthma and immunology	10.1016/j.anai.2022.08.709	Conference presentation
**518**	J. D. J. Spahn, J. S. Hoyte, F. Ambrose, C. S. Martin, N. Vong, S. Caveney, S. Cook, B. Colice, G.	2023	Tezepelumab Efficacy by SNOT-22 Domain Scores in Patients With Severe, Uncontrolled Asthma and Comorbid Nasal Polyps in the Phase 3 Navigator Study	American journal of respiratory and critical care medicine	10.1164/ajrccm-conference.2023.C101	Conference presentation
**521**	M. T. Tabberer, A. Busse, W. Lund, V. Lee, J. Bachert, C. Senior, B. Buchheit, K. Diamant, Z. Sousa, A. et al.,	2021	Mepolizumab reduces disease symptoms for Patients with Chronic Rhinosinusitis with Nasal Polyps: data from the SYNAPSE study	Journal of allergy and clinical immunology	10.1016/j.jaci.2020.12.462	Conference presentation
**546**	S. F. G. Weinstein, M. Bardin, P. Korn, S. Bateman, E. D.	2016	Efficacy of reslizumab with asthma, chronic sinusitis with nasal polyps and elevated blood eosinophils	Journal of allergy and clinical immunology		Conference presentation
**550**	A. F. White, S. Takabayashi, T. Daizadeh, N. Deniz, Y. Rowe, P. Mannent, L. Amin, N. Harel, S. Li, Y. et al.,	2020	Dupilumab effect on type 2 inflammation biomarkers in chronic rhinosinusitis with nasal polyps and NSAID-ERD	Annals of allergy, asthma and immunology	10.1016/j.anai.2020.08.163	Conference presentation
**173**	L. K. De Prado Gomez, A. H. Peters, A. Bachert, C. Wagenmann, M. Heffler, E. Hopkins, C. Hellings, P. Zhang, M. Xing, J. et al.,	2023	EValuating trEatment RESponses of dupilumab versus omalizumab in type 2 patients: the EVEREST trial	Allergy	10.1111/all.15616	Conference presentation

**287**	P. W. P. Hellings, A. Chaker, A. M. Heffler, E. Zhang, H. Daizadeh, N. Nash, S. Khan, A. H. Siddiqui, S. Jacob-Nara, J. A.	2021	Rapid and sustained effects of dupilumab in patients with severe chronic rhinosinusitis with nasal polyps: analysis of the sinus-24 and sinus-52 phase 3 trails	American journal of respiratory and critical care medicine	10.1164/ajrccm-conference.2021.203.1_MeetingAbstracts.A1345	Conference presentation
**352**	T. C. Laidlaw, S. Maspero, J. F. Canonica, G. W. Sher, L. Bachert, C. Han, J. K. Mao, X. Zhang, M. Patel, N. et al.,	2019	Dupilumab improves upper and lower airway outcome measures in patients with severe chronic rhinosinusitis with nasal polyps and comorbid asthma: pooled results from the SINUS-24 and SINUS-52 phase 3 studies	American journal of respiratory and critical care medicine		Conference presentation
**49**	C. H. Bachert, Joseph K Desrosiers, Martin Y Gevaert, Philippe Heffler, Enrico Hopkins, Claire Tversky, Jody R Barker, Peter Cohen, David Emson, Claire	2022	Efficacy and safety of benralizumab in chronic rhinosinusitis with nasal polyps: a randomized, placebo-controlled trial	Journal of Allergy and Clinical Immunology		Duplicate record
**33**	C. H. Bachert, Joseph K Desrosiers, Martin Hellings, Peter W Amin, Nikhil Lee, Stella E Mullol, Joaquim Greos, Leon S Bosso, John V Laidlaw, Tanya M	2019	Efficacy and safety of dupilumab in patients with severe chronic rhinosinusitis with nasal polyps (LIBERTY NP SINUS-24 and LIBERTY NP SINUS-52): results from two multicentre, randomised, double-blind, placebo-controlled, parallel-group phase 3 trials	The Lancet		Duplicate record
**35**	C. H. Bachert, P. W. Mullol, J. Naclerio, R. M. Chao, J. Amin, N. Grabher, A. Swanson, B. N. Hamilton, J. D. Guillonneau, S. Taniou, C. Zhang, D. Pirozzi, G. Graham, N. M. H. Staudinger, H. Mannent, L. P. Khan, A.	2019	Dupilumab improves patient-reported outcomes in patients with chronic rhinosinusitis with nasal polyps and comorbid asthma	J Allergy Clin Immunol Pract	10.1016/j.jaip.2019.03.023	Duplicate record
**41**	C. H. Bachert, P. W. Mullol, J. Hamilos, D. L. Gevaert, P. Naclerio, R. M. Joish, V. N. Chao, J. Mannent, L. P. Amin, N. Abbe, A. Taniou, C. Fan, C. Pirozzi, G. Graham, N. M. H. Mahajan, P. Staudinger, H. Khan, A.	2020	Dupilumab improves health-related quality of life in patients with chronic rhinosinusitis with nasal polyposis	Allergy	10.1111/all.13984	Duplicate record
**57**	C. M. Bachert, L. Naclerio, R. M. Mullol, J. Ferguson, B. J. Gevaert, P. Hellings, P. Jiao, L. Wang, L. Evans, R. R. Pirozzi, G. Graham, N. M. Swanson, B. Hamilton, J. D. Radin, A. Gandhi, N. A. Stahl, N. Yancopoulos, G. D. Sutherland, E. R.	2016	Effect of Subcutaneous Dupilumab on Nasal Polyp Burden in Patients With Chronic Sinusitis and Nasal Polyposis: A Randomized Clinical Trial	Jama	10.1001/jama.2015.19330	Duplicate record
**75**	C. Z. Bachert, S James Hellings, Peter W Mullol, Joaquim Hamilos, Daniel L Gevaert, Philippe Naclerio, Robert M Amin, Nikhil Joish, Vijay N Fan, Chunpeng	2020	Dupilumab reduces opacification across all sinuses and related symptoms in patients with CRSwNP	Rhinology		Duplicate record
**419**	J. L. Mullol, Tanya M Bachert, Claus Mannent, Leda P Canonica, G Walter Han, Joseph K Maspero, Jorge F Picado, Cesar Daizadeh, Nadia Ortiz, Benjamin	2022	Efficacy and safety of dupilumab in patients with uncontrolled severe chronic rhinosinusitis with nasal polyps and a clinical diagnosis of NSAID-ERD: Results from two randomized placebo-controlled phase 3 trials	Allergy		Duplicate record
**112**	A. T. Cakir Cetin, Yesim Keskinoglu, Pembe Arici, Mualla Aylin Onen, Fatos Ecevit, Mustafa Cenk	2023	Methotrexate for recurrent chronic rhinosinusitis with nasal polyps: A randomized, controlled, phase 2 clinical trial	International Forum of Allergy & Rhinology		Irrelevant intervention: DMARD
**386**	E. S. R. Lourijsen, Sietze Vleming, Marleen Hannink, Gerjon Adriaensen, Gwijde FJPM Cornet, Marjolein E Hoven, D Rienk Videler, Ward JM Bretschneider, Jochen H Reinartz, Susanne M	2022	Endoscopic sinus surgery with medical therapy versus medical therapy for chronic rhinosinusitis with nasal polyps: a multicentre, randomised, controlled trial	The Lancet Respiratory Medicine		Irrelevant intervention: steroid
**484**	S. M. L. Ragab, Valerie J Scadding, Glenis Saleh, Hehsam A Khalifa, Mohamed A	2010	Impact of chronic rhinosinusitis therapy on quality of life; A prospective randomized controlled trial	Rhinology		Irrelevant intervention: steroid
**541**	C. Y. Wang, Longgang Chu, Xiaohan Wang, Kuiji Li, Jian Lai, Yinyan Meng, Cuida Wen, Weiping Zhu, Dongdong Zhang, Yuan	2023	Short-term postoperative efficacy of steroid-eluting stents for eosinophilic chronic rhinosinusitis with nasal polyps: A randomized clinical trial	International Forum of Allergy & Rhinology		Irrelevant intervention: steroid

**482**	C. P. G. Price, Amina Stevens, Whitney W Cousens, Leslie Vu, Thanh-Huyen T Suh, Lydia A Erickson, Kristin A Conley, David Grammer, Leslie C Kern, Robert C	2022	Efficacy of an oral CRTH2 antagonist (AZD1981) in the treatment of chronic rhinosinusitis with nasal polyps in adults: A randomized controlled clinical trial	Clinical & Experimental Allergy		Irrelevant intervention: Target therapy
**330**	A. H. A. Khan, A. Falissard, B. Carita, P. Bachert, C. Mullol, J. Reaney, M. Chao, J. Mannent, L. P. Amin, N. et al.,	2021	Data mining of free-text responses: an innovative approach to analyzing patient perspectives on treatment for chronic rhinosinusitis with nasal polyps in a phase iia proof-of-concept study for dupilumab	Patient preference and adherence	10.2147/PPA.S320242	Irrelevant outcome (absenteeism)
**21**	C. C. Bachert, Jonathan Lee, Stella E Zhang, Haixin Harel, Sivan Cunoosamy, Danen Khan, Asif H Jacob-Nara, Juby A Siddiqui, Shahid Nash, Scott	2022	Dupilumab efficacy and biomarkers in chronic rhinosinusitis with nasal polyps: association between dupilumab treatment effect on nasal polyp score and biomarkers of type 2 inflammation in patients with chronic rhinosinusitis with nasal polyps in the phase 3 SINUS-24 and SINUS-52 trials	International Forum of Allergy & Rhinology		Irrelevant outcome (correlation between NPS change and type 2 biomarkers)
**476**	A. T. S. Peters, Zachary M Kern, Robert C Heffler, Enrico Maspero, Jorge F Crampette, Louis Fujieda, Shigeharu Lane, Andrew P Zhang, Haixin Nash, Scott	2022	Improvement in patient-reported “taste” and association with smell in dupilumab-treated patients with severe chronic rhinosinusitis with nasal polyps from the SINUS-24 and SINUS-52 trials	Clinical and Experimental Allergy		Irrelevant outcome (taste)
**128**	R. K. Chan, Chris RuiWen Lipworth, Brian	2020	Disconnect between effects of mepolizumab on severe eosinophilic asthma and chronic rhinosinusitis with nasal polyps	The Journal of Allergy and Clinical Immunology: In Practice		Letter to Editor
**501**	J. Schneider	2016	Subcutaneous dupilumab and mometasone furoate nasal spray for chronic rhinosinusitis with polyps	JAMA otolaryngology-head & neck surgery	10.1001/jamaoto.2016.0678	Letter to Editor
**511**	S. B. Siddiqui, C. Chaker, A. M. Han, J. K. Hellings, P. W. Peters, A. T. Heffler, E. Kamat, S. Zhang, H. Nash, S. Khan, A. H. De Prado Gomez, L. Jacob-Nara, J. A. Rowe, P. J. Deniz, Y.	2022	AROMA: real-world global registry of dupilumab for chronic rhinosinusitis with nasal polyps	ERJ Open Res	10.1183/23120541.00085-2022	Letter to Editor
**280**	T. W. C. Harrison, P. Menzella, F. Canonica, G. W. Louis, R. Cosio, B. G. Lugogo, N. L. Mohan, A. Burden, A. McDermott, L. et al.,	2021	Onset of effect and impact on health-related quality of life, exacerbation rate, lung function, and nasal polyposis symptoms for patients with severe eosinophilic asthma treated with benralizumab (ANDHI): a randomised, controlled, phase 3b trial	Lancet respiratory medicine	10.1016/S2213-2600(20)30414-8	No data on CRSwNP
**43**	C. H. Bachert, P. Lund, V. Mayer, B. Chan, R. H. Smith, S. G. Sousa, A. R. Alfonso-Cristancho, R. Yang, S.	2021	Improving quality of life and productivity in patients with chronic rhinosinusitis with nasal polyps treated with mepolizumab: SF-36 and WPAI results from SYNAPSE	Allergy	10.1111/all.15098	Not found
**48**	C. H. Bachert, C. L. Han, J. K. Fokkens, W. J. Nash, S. Khan, A. H.	2022	Symptom-Free Days in Patients With Severe CRSwNP Treated With Dupilumab	Otolaryngology - head and neck surgery	10.1177/01945998221107672	Not found
**106**	G. G. G. Brusselle, Philippe	2021	Mepolizumab for chronic rhinosinusitis with nasal polyps	The Lancet Respiratory medicine		Not found
**415**	J. B. Mullol, C. Fokkens, W. Smith, S. G. Keeley, T. Mayer, B. Howarth, P. Chan, R. Yancey, S.	2023	The impact of mepolizumab therapy on sleep in patients with chronic rhinosinusitis with nasal polyps: data from the SYNAPSE study	Allergy	10.1111/all.15616	Not found
**6**	T. S. Albrecht, Martin M Capitani, Flavia van Schaik, Carolina Löwenheim, Hubert Becker, Sven	2023	Real-world evidence for the effectiveness and safety of dupilumab in patients with CRSwNP after 1 year of therapy	World Allergy Organization Journal		NRSI
**22**	C. D. Bachert, M. Mullol, J. Maspero, J. F. Wagenmann, M. Niemann, I. Khan, A. Kamat, S. Amin, N. Mannent, L. P.	2019	Baseline Characteristics of Patients With Chronic Rhinosinusitis With Nasal Polyps (With and Without Asthma) Enrolled in SINUS-52, a Randomized, Double-Blind, Phase 3 Study of Dupilumab	Laryngo- rhino- otologie		NRSI
**27**	C. G. Bachert, P. Corren, J. Mullol, J. Han, J. Ow, R. Hussain, I. Islam, L. Fogel, R. Kaufman, D. et al.,	2019	Baseline characteristics of phase 3 randomized controlled trials of omalizumab in chronic rhinosinusitis with nasal polyps	Allergy	10.1111/all.13959	NRSI

**87**	T. S. Bidder, Jagdeep Rennie, Catherine Lund, Valerie J Robinson, Douglas S Kariyawasam, Harsha H	2018	Omalizumab treats chronic rhinosinusitis with nasal polyps and asthma together-a real life study	Rhinology		NRSI
**95**	R. E. Book, Shalom Tal, Yuval Eliashar, Ron	2023	Biological Treatment for Uncontrolled Chronic Rhinosinusitis with Nasal Polyps: Preliminary Real-World Results from a Tertiary Medical Center	Journal of Clinical Medicine		NRSI
**107**	W. W. P. Busse, I. D. Siddiqui, S. Khan, A. H. Praestgaard, A. Nash, S. Jacob-Nara, J. A. Rowe, P. J. Deniz, Y.	2023	Dupilumab Improves Outcomes in Patients with Chronic Rhinosinusitis with Nasal Polyps and Coexisting Asthma Irrespective of Baseline Asthma Characteristics	Journal of asthma and allergy	10.2147/JAA.S391896	NRSI
**121**	E. D. C. Cantone, Eugenio Ricciardiello, Filippo Di Nola, Claudio Grimaldi, Giusi Allocca, Viviana Motta, Gaetano	2022	Olfaction recovery following dupilumab is independent of nasal polyp reduction in CRSwNP	Journal of Personalized Medicine		NRSI
**166**	Z. L. Danisman, Maximilian Kühn, Jan Philipp Linxweiler, Barbara Solomayer, Erich-Franz Wagner, Mathias Wagenpfeil, Gudrun Schick, Bernhard Berndt, Sabrina	2023	Differential nasal swab cytology represents a valuable tool for therapy monitoring but not prediction of therapy response in chronic rhinosinusitis with nasal polyps treated with Dupilumab	Frontiers in immunology		NRSI
**170**	E. F. De Corso, Gianluca Salsi, Daria Fanelli, Francesca Ronci, Gianluca Sala, Giovanna Bitonti, Rossella Cuda, Domenico	2022	Cost–utility analysis of dupilumab for the treatment of chronic rhinosinusitis with nasal polyps (CRSwNP) in Italy	Journal of Personalized Medicine		NRSI
**171**	E. P. De Corso, Ernesto Trimarchi, Matteo La Mantia, Ignazio Pagella, Fabio Ottaviano, Giancarlo Garzaro, Massimiliano Pipolo, Carlotta Torretta, Sara Seccia, Veronica	2023	Dupilumab in the treatment of severe uncontrolled chronic rhinosinusitis with nasal polyps (CRSwNP): A multicentric observational Phase IV real-life study (DUPIREAL)	Allergy		NRSI
**188**	A. T. Detoraki, Eugenio D'Amato, Maria Calabrese, Cecilia Casella, Claudia Maniscalco, Mauro Poto, Remo Brancaccio, Raffaele Boccia, Matilde Martino, Maria	2021	Mepolizumab improves sino-nasal symptoms and asthma control in severe eosinophilic asthma patients with chronic rhinosinusitis and nasal polyps: a 12-month real-life study	Therapeutic Advances in Respiratory Disease		NRSI
**189**	H. F. Dharmarajan, Oluleke Lee, Stella E Wang, Eric W	2022	Outcomes of dupilumab treatment versus endoscopic sinus surgery for chronic rhinosinusitis with nasal polyps	International Forum of Allergy & Rhinology		NRSI
**213**	S. M. Ferri, Carlo Casini, Marta Malvezzi, Luca Pirola, Francesca Russo, Elena Racca, Francesca Messina, Maria Rita Puggioni, Francesca Nappi, Emanuele	2024	Sleep quality burden in chronic rhinosinusitis with nasal polyps and its modulation by dupilumab	Annals of Allergy, Asthma & Immunology		NRSI
**231**	S. C. Gallo, Paolo Spirito, Luca Feduzi, Marta Seccia, Veronica Visca, Dina Spanevello, Antonio Statuti, Erica Latorre, Manuela Montuori, Claudio	2022	Mepolizumab improves outcomes of chronic rhinosinusitis with nasal polyps in severe asthmatic patients: a multicentric real-life study	Journal of personalized medicine		NRSI
**232**	E. N. Garvey, Bita Duffy, Alexander Hannikainen, Paavali Kahn, Chase Farquhar, Douglas Rosen, Marc Rabinowitz, Mindy Toskala, Elina Nyquist, Gurston	2024	Optimizing the timing of biologic and surgical therapy for patients with refractory chronic rhinosinusitis with nasal polyposis (CRSwNP)	International Forum of Allergy & Rhinology		NRSI
**237**	K. K. Gerstacker, Manuel Christoph Jakob, Till Fabian Hildenbrand, Tanja	2023	Real Life Observational Study of Treatment Success of Monoclonal Antibodies for Refractory Chronic Rhinosinusitis with Nasal Polyps	Journal of Clinical Medicine		NRSI
**255**	F. P. Giombi, G. M. Nappi, E. Giunta, G. Muci, G. Pirola, F. Ferreli, F. Heffler, E. Paoletti, G. Giannitto, C. Mercante, G. Francone, M. Spriano, G. Canonica, G. W. Malvezzi, L.	2024	Radiological Versus Clinical 1-Year Outcomes of Dupilumab in Refractory CRSwNP: A Real-Life Study	Laryngoscope	10.1002/lary.31238	NRSI
**317**	F. B. Jansen, Benjamin Eden, Jördis K Breda, Philippe C Hot, Amra Oqueka, Tim Betz, Christian S Hoffmann, Anna S	2023	Dupilumab (Dupixent®) tends to be an effective therapy for uncontrolled severe chronic rhinosinusitis with nasal polyps: Real data of a single-centered, retrospective single-arm longitudinal study from a university hospital in Germany	European Archives of Oto-Rhino-Laryngology		NRSI
**351**	I. O. La Mantia, Giancarlo Ragusa, Martina Trimarchi, Matteo Foglia, Emanuela	2024	Multidimensional Impact of Dupilumab on Chronic Rhinosinusitis with Nasal Polyps: A Complete Health	Journal of Personalized Medicine		NRSI

	Schettini, Fabrizio Bellavia, Daniele Cantone, Elena		Technology Assessment of Clinical, Economic, and Non-Clinical Domains			
**360**	T. M. M. Laidlaw, Joaquim Fan, Chunpeng Zhang, Donghui Amin, Nikhil Khan, Asif Chao, Jingdong Mannent, Leda P	2019	Dupilumab improves nasal polyp burden and asthma control in patients with CRSwNP and AERD	The Journal of Allergy and Clinical Immunology: In Practice		NRSI
**397**	T. T. Matsuyama, Hideyuki Tada, Hiroe Chikamatsu, Kazuaki	2023	Circulating T cell subsets and ILC2s are altered in patients with chronic rhinosinusitis with nasal polyps after dupilumab treatment	American Journal of Rhinology & Allergy		NRSI
**399**	J. C.-H. Maza-Solano, C. Alobid, I. Álvarez-Cendrero, M. Palomares, Ó Moreno-Luna, R. Santos-Perez, J. González-García, J. Sánchez-Gómez, S.	2022	Nasal Symptoms in Asthmatic Patients under Treatment with Anti-IL-5 Monoclonal Antibodies. A Real-Life Cohort Study	J Clin Med	10.3390/jcm11237056	NRSI
**401**	J. C.-L. Maza-Solano, A. Martin-Jimenez, D. Moreno-Luna, R. Gonzalez-Garcia, J. Cuvillo, A. Sanchez-Gomez, S.	2023	Omalizumab Treatment in Uncontrolled Asthma and CRSwNP Patients, with Previous Endoscopic Sinus Surgery, to Improve Quality of Life and Endoscopic Outcomes: a Two-Year Real-Life Study	Curr Allergy Asthma Rep	10.1007/s11882-023-01106-w	NRSI
**402**	V. M. H. McDonald, Y. Agusti, A. Gibson, P. G.	2024	Treatable Traits in Asthma: The Importance of Extrapulmonary Traits—GERD, CRSwNP, Atopic Dermatitis, and Depression/Anxiety	Journal of Allergy and Clinical Immunology: In Practice	10.1016/j.jaip.2024.01.020	NRSI
**403**	E. C. S.-G. Meier, Peter Steiner, Urs C Soyka, Michael B	2021	Real-life experience of monoclonal antibody treatments in chronic rhinosinusitis with nasal polyposis	International Archives of Allergy and Immunology		NRSI
**454**	E. B. Nettis, Luisa Patella, Vincenzo Bonzano, Laura Detoraki, Aikaterini Di Leo, Elisabetta Sirufo, Maria Maddalena Caruso, Cristiano Lodi Rizzini, Fabio Conte, Mariaelisabetta	2022	Effectiveness and safety of dupilumab in patients with chronic rhinosinusitis with nasal polyps and associated comorbidities: a multicentric prospective study in real life	Clinical and Molecular Allergy		NRSI
**456**	S. C. Nolasco, Claudia Pelaia, Corrado Benfante, Alida Caiaffa, Maria Filomena Calabrese, Cecilia Carpagnano, Giovanna Elisiana Ciotta, Domenico D'Amato, Maria Macchia, Luigi	2021	Benralizumab effectiveness in severe eosinophilic asthma with and without chronic rhinosinusitis with nasal polyps: a real-world multicenter study	The Journal of Allergy and Clinical Immunology: In Practice		NRSi
**461**	P. L. Orlando, Giuseppe Kuitche, Donald Matucci, Andrea Vultaggio, Alessandra Gallo, Oreste Maggiore, Giandomenico	2024	Effectiveness of dupilumab versus endoscopic sinus surgery for the treatment of type-2 chronic rhinosinusitis with nasal polyps: a preliminary report	European Archives of Oto-Rhino-Laryngology		NRSI
**469**	G. P. Pecorari, Federica Borgione, Mario Prizio, Carmine Della Mantica, Gregorio Galli Garetto, Marco Gedda, Francesco Riva, Giuseppe	2023	The role of intranasal corticosteroids in chronic rhinosinusitis with nasal polyposis treated with dupilumab	American Journal of Otolaryngology		NRSI
**470**	C. B. Pelaia, Alida Busceti, Maria Teresa Caiaffa, Maria Filomena Campisi, Raffaele Carpagnano, Giovanna Elisiana Crimi, Nunzio D'Amato, Maria Foschino Barbaro, Maria Pia Maglio, Angelantonio	2023	Real-life effects of dupilumab in patients with severe type 2 asthma, according to atopic trait and presence of chronic rhinosinusitis with nasal polyps	Frontiers in Immunology		NRSI
**499**	G. A. W. Scangas, Arthur W Ting, Jonathan Y Metson, Ralph Walgama, Evan Shrime, Mark G Higgins, Thomas S	2021	Cost utility analysis of dupilumab versus endoscopic sinus surgery for chronic rhinosinusitis with nasal polyps	The Laryngoscope		NRSI
**520**	A. R. K. Swisher, Rijul S Vu, Priscilla Q Liang, Jonathan	2023	Ocular Surface Adverse Events are not Associated with Dupilumab use in Nasal Polyp Treatment	The Laryngoscope		NRSI
**545**	M. E. K. Wechsler, Amy D Paggiaro, Pierluigi Nair, Parameswaran Staumont-Salle, Delphine Radwan, Amr Johnson, Robert R Kapoor, Upender Khokhar, Faisal A Daizadeh, Nadia	2022	Effect of dupilumab on blood eosinophil counts in patients with asthma, chronic rhinosinusitis with nasal polyps, atopic dermatitis, or eosinophilic esophagitis	The Journal of Allergy and Clinical Immunology: In Practice		NRSI
**562**	I. T. Yilmaz, Murat Bahçecioğlu, Sakine Nazik Tutar, Nuri Gülmez, İnci	2020	Efficacy of mepolizumab treatment in oral corticosteroid-dependent severe eosinophilicasthma patients with	Turkish Journal of Medical Sciences		NRSI

			chronic rhinosinusitis with nasal polyps: single center, real life study			
**350**	I. G. La Mantia, Egle Ragusa, Martina Cocuzza, Salvatore Radulesco, Thomas Saibene, Alberto Maria Calvo-Henriquez, Christian Fakhry, Nicolas Michel, Justin Maniaci, Antonino	2024	Effectiveness and rapidity on olfatory fuction recovery in CRS patients treated with Dupilumab: A real life prospective controlled study	European Archives of Oto-Rhino-Laryngology		NRSI
**180**	E. V. Z. De Schryver, T. Bachert, C. Gevaert, P.	2015	Comparison of different medical treatment options for CRSwNP: doxycycline, methylprednisolone, mepolizumab and omalizumab	Allergy	10.1111/all.12720	Conference presentation
**200**	B. E. Euctr	2017	A Phase 3 Clinical Trial of Omalizumab for Chronic Rhinosinusitus with Nasal Polyps	https://trialsearch.who.int/Trial2.aspx?TrialID=EUCTR2017-001718-28-BE		Protocol without results
**202**	C. Z. Euctr	2017	A Phase 3 Clinical Trial of Omalizumab for Chronic Rhinosinusitus with Nasal Polyps	https://trialsearch.who.int/Trial2.aspx?TrialID=EUCTR2017-001724-22-CZ		Protocol without results
**203**	D. E. Euctr	2015	Effects of mepolizumab compared to placebo on airway physiology in patients with eosinophilic asthma: MEMORY study	https://trialsearch.who.int/Trial2.aspx?TrialID=EUCTR2015-001868-19-DE		Protocol without results
**204**	D. K. Euctr	2020	Efficacy and safety of tezepelumab in participants with Severe Chronic Rhinosinusitis with Nasal Polyposis	https://trialsearch.who.int/Trial2.aspx?TrialID=EUCTR2020-003062-39-DK		Protocol without results
**205**	F. I. Euctr	2017	A Phase 3 Clinical Trial of Omalizumab for Chronic Rhinosinusitus with Nasal Polyps	https://trialsearch.who.int/Trial2.aspx?TrialID=EUCTR2017-001718-28-FI		Protocol without results
**206**	G. B. Euctr	2016	A Phase 2 Study of the Safety and Efficacy of AK001 in Patients with Nasal Polyps	https://trialsearch.who.int/Trial2.aspx?TrialID=EUCTR2016-000460-42-GB		Protocol without results
**207**	H. U. Euctr	2020	Efficacy and safety of benralizumab for prevention of nasal polyps recurrence following endoscopic sino-nasal surgery	https://trialsearch.who.int/Trial2.aspx?TrialID=EUCTR2020-000195-38-HU		Protocol without results
**209**	H. U. Euctr	2021	A study to evaluate the efficacy and safety of repeating dose of benralizumab administered SC versus placebo in patients with Eosinophilic Chronic Rhinosinusitis with Nasal Polyps	https://trialsearch.who.int/Trial2.aspx?TrialID=EUCTR2021-000267-72-HU		Protocol without results
**211**	P. L. Euctr	2017	A Phase 3 Clinical Trial of Omalizumab for Chronic Rhinosinusitus with Nasal Polyps	https://trialsearch.who.int/Trial2.aspx?TrialID=EUCTR2017-001724-22-PL		Protocol without results
**315**	Irct20220218054057N	2023	Omalizumab Efficacy in Chronic Rhinosinusitis Patients with Recurrent Nasal Polyp	https://trialsearch.who.int/Trial2.aspx?TrialID=IRCT20220218054057N1		Protocol without results
**322**	jRct	2021	Efficacy and Safety of Mepolizumab in Adults With Chronic Rhinosinusitis With Nasal Polyps (CRSwNP)/ Eosinophilic Chronic Rhinosinusitis (ECRS) (MERIT)	https://rctportal.niph.go.jp/en/detail?trial_id=jRCT2031200306		Protocol without results
**526**	Tctr	2019	A Multicentre, Randomised, Double-Blind, Parallel-Group, Placebo- Controlled Phase 3 Efficacy and Safety Study of Benralizumab in Patients with Eosinophilic Chronic Rhinosinusitis with Nasal Polyps (ORCHID)	https://trialsearch.who.int/Trial2.aspx?TrialID=TCTR20190820003		Protocol without results
**199**	B. E. Euctr	2013	A Randomized, Double-Blind, Phase 2, Placebo Controlled, 2 Arm Study To Evaluate Dupilumab In Patients With Bilateral Nasal Polyposis And Chronic Symptoms Of Sinusitis	EUCTR [www.clinicaltrialsregister.eu]		Protocol without results
**197**	W. S. Eschenbacher, Matthew Knoeddler, Alice Li, Rung-chi Borish, Larry	2020	Biologics for the treatment of allergic rhinitis, chronic rhinosinusitis, and nasal polyposis	Immunology and allergy clinics of North America		Review article
**212**	V. C. Favier, J. Crampette, L. Bourdin, A. Ahmed, E.	2023	What place will tezepelumab hold in the treatment paradigm in chronic rhinosinusitis?	Expert Review of Clinical Immunology	10.1080/1744666X.2023.2215986	Review article
**223**	W. V. D. L. Fokkens, Rik Reitsma, Sietze	2021	Dupilumab for the treatment of chronic rhinosinusitis with nasal polyposis	Expert Opinion on Biological Therapy		Review article
**406**	A. S. Miglani, Zachary M Smith, Timothy L Mace, Jess C Schlosser, Rodney J	2023	A comparative analysis of endoscopic sinus surgery versus biologics for treatment of chronic rhinosinusitis with nasal polyposis	International Forum of Allergy & Rhinology		Review article

**472**	A. T. H. Peters, Joseph K Hellings, Peter Heffler, Enrico Gevaert, Philippe Bachert, Claus Xu, Yingxin Chuang, Chien-Chia Neupane, Binod Msihid, Jérôme	2021	Indirect treatment comparison of biologics in chronic rhinosinusitis with nasal polyps	The Journal of Allergy and Clinical Immunology: In Practice	Review article
**430**	Nct	2013	An Evaluation of Dupilumab in Patients With Nasal Polyposis And Chronic Symptoms Of Sinusitis	https://clinicaltrials.gov/show/NCT01920893	Study protocol
**431**	Nct	2013	A Randomized, Double-Blind, Phase 2, Placebo Controlled, 2 Arm Study To Evaluate Dupilumab In Patients With Bilateral Nasal Polyposis And Chronic Symptoms Of Sinusitis	Clinicaltrials.gov [www.clinicaltrials.gov]	Study protocol
**432**	Nct	2017	Benralizumab Effect on Severe Chronic Rhinosinusitis With Eosinophilic Polyposis	https://clinicaltrials.gov/show/NCT03450083	Study protocol
**433**	Nct	2017	A clinical trial of omalizumab in participants with chronic rhinosinusitis with nasal polyps	https://clinicaltrials.gov/show/NCT03280550	Study protocol
**435**	Nct	2018	Etokimab in Adults With Chronic Rhinosinusitis With Nasal Polyps (CRSwNP)	https://clinicaltrials.gov/show/NCT03614923	Study protocol
**436**	Nct	2018	Effect of Benralizumab in Patients With Severe Nasal Polyps	https://clinicaltrials.gov/show/NCT03627286	Study protocol
**437**	Nct	2018	An Extension Study of Omalizumab in Participants With Chronic Rhinosinusitis With Nasal Polyps	https://clinicaltrials.gov/show/NCT03478930	Study protocol
**438**	Nct	2019	Efficacy and Safety Study of Benralizumab in Patient With Eosinophilic Chronic Rhinosinusitis With Nasal Polyps (ORCHID)	https://clinicaltrials.gov/ct2/show/NCT04157335	Study protocol
**439**	Nct	2020	Efficacy of Dupilumab for Patients With Chronic Rhinosinusitis Without Nasal Polyps (CRSsNP)	https://clinicaltrials.gov/show/NCT04362501	Study protocol
**440**	Nct	2020	Efficacy and Safety of Mepolizumab in Adults With Chronic Rhinosinusitis With Nasal Polyps (CRSwNP)/ Eosinophilic Chronic Rhinosinusitis (ECRS)	https://clinicaltrials.gov/ct2/show/NCT04607005	Study protocol
**442**	Nct	2021	EValuating trEatment RESponses of Dupilumab Versus Omalizumab in Type 2 Patients	https://clinicaltrials.gov/ct2/show/NCT04998604	Study protocol
**443**	Nct	2021	Efficacy and Safety of Tezepelumab in Participants With Severe Chronic Rhinosinusitis With Nasal Polyposis	https://clinicaltrials.gov/ct2/show/NCT04851964	Study protocol
**444**	Nct	2022	Omalizumab Efficacy in Patients With Refractory Nasal Polyps	https://clinicaltrials.gov/show/NCT05405478	Study protocol
**445**	Nct	2022	Comparison of Immune Profiles in Chronic Rhinosinusitis Patients After Mepolizumab Treatment	https://clinicaltrials.gov/ct2/show/NCT05642806	Study protocol
**446**	Nct	2022	Mepolizumab to CRSwNP Through 12 Months - Randomised to FESS and Non-FESS Within the First 2 Weeks	https://clinicaltrials.gov/show/NCT05598814	Study protocol
**447**	Nct	2022	Add-on Dupilumab for AFRS as Postoperative Therapy (ADAPT)	https://clinicaltrials.gov/show/NCT05545072	Study protocol
**448**	Nct	2023	Dupilumab in Chinese Adult Participants With CRSwNP	https://clinicaltrials.gov/ct2/show/NCT05878093	Study protocol
**449**	Nct	2023	A Randomized, Real-world Head-to-head Study of Dupilumab Versus Mepolizumab in Danish CRSwNP Patients	https://clinicaltrials.gov/ct2/show/NCT05942222	Study protocol
**450**	Nct	2023	Mepolizumab and In-office Nasal Polypectomy in Patients With Chronic Rhinosinusitis (CRS). A Three Arm Study	https://clinicaltrials.gov/show/NCT05923047	Study protocol
**451**	Nct	2024	A Study of Lebrikizumab (LY3650150) in Adult Participants With Chronic Rhinosinusitis and Nasal Polyps Treated With Intranasal Corticosteroids (CONTRAST-NP)	https://clinicaltrials.gov/ct2/show/NCT06338995	Study protocol
**160**	Ctis	2023	Biologic treatment of rhinosinusitis with nasal polyposis in real-world Danish patients - a direct comparison of dupilumab and mepolizumab	https://trialsearch.who.int/Trial2.aspx?TrialID=CTIS2022-502250-14-00	Study protocol (without results)

**161**	Ctis	2023	A phase IV, randomized double blinded placebo-controlled study on the barrier restoring capacity of Dupilumab in type 2 airway disease: cRSwNP	https://trialsearch.who.int/Trial2.aspx?TrialID=CTIS2023-504014-30-00		Study protocol (without results)
**162**	Ctri	2022	Study assessing the efficacy and safety of dupilumab in patients with Allergic Fungal Rhinosinusitis (AFRS)	https://trialsearch.who.int/Trial2.aspx?TrialID=CTRI/2022/05/042593		Study protocol (without results)
**477**	A. T. W. Peters, M. Bernstein, J. A. Khan, A. H. Nash, S. Jacob-Nara, J. A. Siddiqui, S. Rowe, P. J. Deniz, Y.	2023	Dupilumab efficacy in patients with chronic rhinosinusitis with nasal polyps with and without allergic rhinitis	Allergy and asthma proceedings	10.2500/aap.2023.44.230015	Subgroup analysis based on allergic rhinitis status at baseline
**68**	C. S. Bachert, A. R. Han, J. K. Schlosser, R. J. Sowerby, L. J. Hopkins, C. Maspero, J. F. Smith, S. G. Kante, O. Karidi-Andrioti, D. E. et al.,	2022	Mepolizumab for chronic rhinosinusitis with nasal polyps: treatment efficacy by comorbidity and blood eosinophil count	Journal of allergy and clinical immunology	10.1016/j.jaci.2021.10.040	Subgroup analysis based on asthma comorbidity and eosinophil count at baseline
**354**	T. M. B. Laidlaw, C. Amin, N. Desrosiers, M. Hellings, P. W. Mullol, J. Maspero, J. F. Gevaert, P. Zhang, M. Mao, X. et al.,	2021	Dupilumab improves upper and lower airway disease control in chronic rhinosinusitis with nasal polyps and asthma	Annals of allergy, asthma & immunology	10.1016/j.anai.2021.01.012	Subgroup analysis based on asthma status at baseline
**300**	C. M. Hopkins, J. Khan, A. H. Lee, S. E. Wagenmann, M. Hellings, P. Fokkens, W. Msihid, J. Nair, R. Kamat, S. et al.,	2024	Impact of Dupilumab on Sinonasal Symptoms and Outcomes in Severe Chronic Rhinosinusitis With Nasal Polyps	Otolaryngology--head and neck surgery	10.1002/ohn.627	Subgroup analysis based on CRSwNP severity at baseline
**417**	J. L. Mullol, T. M. Bachert, C. Mannent, L. P. Canonica, G. W. Han, J. K. Maspero, J. F. Picado, C. Daizadeh, N. Ortiz, B. et al.,	2022	Efficacy and safety of dupilumab in patients with uncontrolled severe chronic rhinosinusitis with nasal polyps and a clinical diagnosis of NSAID-ERD: results from two randomized placebo-controlled phase 3 trials	Allergy	10.1111/all.15067	Subgroup analysis based on NSAID-ERD
**394**	J. F. B. Maspero, Claus Martinez, Fernando J Hanania, Nicola A Ortiz, Benjamin Patel, Naimish Mannent, Leda P Praestgaard, Amy Pandit-Abid, Nami Siddiqui, Shahid	2023	Clinical efficacy among patients with chronic rhinosinusitis with nasal polyps and clinical features of obstructive lung disease: post hoc analysis of the Phase III SINUS-24 and SINUS-52 studies	Journal of Asthma and Allergy		Subgroup analysis based on obstructive lung disease at baseline
**303**	C. W. Hopkins, M. Bachert, C. Desrosiers, M. Han, J. K. Hellings, P. W. Lee, S. E. Msihid, J. Radwan, A. Rowe, P. et al.,	2021	Efficacy of dupilumab in patients with a history of prior sinus surgery for chronic rhinosinusitis with nasal polyps	International forum of allergy & rhinology	10.1002/alr.22780	Subgroup analysis based on prior NP surgery
**214**	W. J. M. Fokkens, J. Kennedy, D. Philpott, C. Seccia, V. Kern, R. C. Coste, A. Sousa, A. R. Howarth, P. H. Benson, V. S. et al.,	2023	Mepolizumab for chronic rhinosinusitis with nasal polyps (SYNAPSE): in-depth sinus surgery analysis	Allergy	10.1111/all.15434	Subgroup analysis based on timing of prior NP surgery
**225**	S. M. Fujieda, Shoji Takeno, Sachio Asako, Mikiya Takeuchi, Makiko Fujita, Hiroyuki Takahashi, Yoshinori Amin, Nikhil Deniz, Yamo Rowe, Paul	2021	The effect of dupilumab on intractable chronic rhinosinusitis with nasal polyps in Japan	The Laryngoscope		Subgroup analysis of a single center (Japan)

YOP: Year of Publication; CRSwNP: Chronic Rhinosinusitis with Nasal Polyps.

Table S3. A list of outcomes reported by RCTs comparing various biological therapies in CRSwNP not eligible for meta-analysis

Author (YOP)	Trial (Biological Therapy)	Sample (Intervention/control)	Outcome	Timepoint	Effect measure	
					**Intervention**	**Placebo**
**Weinstein (2019)**	BREATHE (Reslizumab)	78 / 72	Clinical asthma exacerbation requiring SCS for 3 days or more	52 weeks	no. (%) = 0.397 (0.005%)	no. (%) = 2.23 (0.031%)
			Improvement exceeding MCID for ACQ-6	52 weeks	no. (%) = 59 (88.05%)	no. (%) = 39 (63.93%)
			Improvement exceeding MCID for AQLQ	52 weeks	no. (%) = 59 (86.76%)	no. (%) = 41 (66.12%)
**Canonica (2022)**	NCT03170271 (Benralizumab)	96 / 57	No asthma exacerbation	24 weeks	N/T = 74/96	N/T = 21/57
			Change in SGRQ from baseline	24 weeks	Mean = -28.5	Mean = -11.8
**Zhang (2023)**	CROWNS-1 (CM310)	28 / 28	Change in tissue Eosinophil % from baseline	16 weeks	Mean (SD) = -27 (34)	Mean (SD) = -7 (33)
			% of sinus inflammation volume occupied by the disease (whole nasal cavity)	16 weeks	Mean (SD) = -39 (19)	Mean (SD) = -2 (20)
			% of sinus inflammation volume occupied by the disease (frontal sinus)	16 weeks	Mean (SD) = -38 (40)	Mean (SD) = 5 (23)
			% of sinus inflammation volume occupied by the disease (ethmoidal sinus)	16 weeks	Mean (SD) = -48 (28)	Mean (SD) = -3 (20)
			% of sinus inflammation volume occupied by the disease (sphenoidal sinus)	16 weeks	Mean (SD) = - 50 (24)	Mean (SD) = -9 (37)
			% of sinus inflammation volume occupied by the disease (maxillary sinus)	16 weeks	Mean (SD) = -31 (26)	Mean (SD) = -2 (24)
**Laidlaw (2023)**	NAVIGATORY (Tezepelumab)	62 / 56	Change in ASD from baseline	52 weeks	Mean (SD) = -0.8 (0.078)	Mean (SD) = -0.35 (0.085)
			Change in FeNO (ppb) from baseline	52 weeks	Mean = 0.58	Mean = 0.99
			Chane in IL-13 (pg/mL) from baseline	52 weeks	Mean = 0.45	Mean = 0.95
			Change in EDN (Mg/L) from baseline	52 weeks	Mean = 0.66	Mean = 1.07
			Change in MMP-10 (Mg/L) from baseline	52 weeks	Mean = 0.78	Mean = 1.01
			Change in tissue IL6 (pg/mL) from baseline	52 weeks	Mean = 1.03	Mean = 1.02
			Change in IL-8 (pg/mL) from baseline	52 weeks	Mean = 1.06	Mean = 1.11
**Gevaert (2013)**	- (Omalizumab)	7 / 7	Change in SF-36 (mental) from baseline	16 weeks	Mean = 8.76	N/A
			Change in SF-36 (physical) from baseline	16 weeks	Mean = -0.3	N/A
**Wahba (2019)**	- (Omalizumab)	43 / 43	Disease recurrence	16 weeks	N/T = 19/43	N/T = 8/43
**Tversky (2021)**	NCT03450083 (Benralizumab)	12 / 12	Change in NBS score from baseline	20 weeks	Mean (SD) = -1.4 (0.4)	Mean (SD) = -0.3 (1.1)
**Gevaert (2011)**	CRT110178 (Mepolizumab)	20 / 20	Change in tissue IL-6 (pg/mL) from baseline	8 weeks	Mean = 0.44	Mean = 0.88
			Change in tissue MPO from baseline	4 weeks	Mean = -2830	Mean = 8995
				8 weeks	Mean = -6130	Mean = 10030
			Change in tissue IL-1b from baseline	4 weeks	Mean = 0.45	Mean = 0.9
				8 weeks	Mean = 0.51	Mean = 0.93

**Schryver (2017)**	Omalizumab vs. Mepolizumab	28 / 34	Recurrence rate	4 weeks	N/T = 13/28	N/T = 14/34
				8 weeks		
**Bachert (2022)**	OSTRO (Benralizumab)	207 / 203	Change in NBS score from baseline	40 weeks	Mean = -0.73	Mean = -0.41
				56 weeks	Mean = -0.7	Mean = -0.42
**Emson (2024)**	OSTRO (Benralizumab)	207 / 203	Change in EDN (Mg/L) from baseline	24 weeks	Mean = -300	Mean = -220
				56 weeks	Mean = -300	Mean = -220
**Gevaert (2020)**	POLYP1 (Omalizumab)	72 / 66	LS mean change in LoS – VAS from baseline	24 weeks	Mean (SD) = -0.56 (0.09)	Mean (SD) = -0.23 (0.1)
	POLYP2 (Omalizumab)	62 / 65		24 weeks	Mean (SD) = -0.58 (0.1)	Mean (SD) = -0.13 (0.1)
			LS mean change in postnasal drip – VAS from baseline	24 weeks	Mean (SD) = -0.72 (0.1)	Mean (SD) = -0.16 (0.1)
				24 weeks	Mean (SD) = -0.55 (0.1)	Mean (SD) = 0.0 (0.1)
			LS mean change in runny nose – VAS from baseline	24 weeks	Mean (SD) = -0.77 (0.1)	Mean (SD) = -0.34 (0.1)
				24 weeks	Mean (SD) = -0.7 (0.1)	Mean (SD) = -0.08 (0.1)
**Han (2021)**	SYNAPSE (Mepolizumab)	206 / 201	LS mean change in LoS – VAS from baseline	52 weeks	Mean (SD) = -2.8 (3.61)	Mean (SD) = -1.4 (2.65)

LoS: loss of smell; SD: standard deviation; LS: least square; VAS: visual analogue scale; MCID: Minimal clinically important difference; FeNO: fractional exhaled Nitric Oxide; EDN: Eosinophil-derived neurotoxin; MMP-10: Matrix metalloproteinase; LS: least-squares; ECP: Eosinophil Cationic Protein; TNSS: Total Nasal Symptom Score.

Table S4. The number of patients analyzed in each outcome analysis

Drug	NCS (cont)	NPS (cont)	UPSIT	TNSS	Lund	NP Surgery	SCS Use	ACQ-6	AQLQ	SNOT	VAS-rhinorrhea	VAS-loss of smell	VAS-nasal symptoms	AEs
**Benralizumab**	-	195	-	-	115	207	154	93	-	301	-	21	21	325
**CM310**	28	28	28	28	28	-	-	-	-	28	-	-	-	28
**Dupilumab**	150	166	311	-	159	609	233	355	-	342	16	432	289	470
**Mepolizumab**	-	433	203	-	104	259	259	-	-	401	268	558	215	429
**Omalizumab**	195	253	207	238	50	129	129	-	161	195	15	200	15	266
**PF-06817024**	-	-	-	-	-	-	-	-	-	-	-	-	-	11
**Placebo**	578	1255	699	263	638	1191	941	414	268	1373	269	1317	448	1501
**Tezepelumab**	203	203	-	-	203	203	203	57	62	254	-	203	-	203
**Reslizumab**	-	8	-	-	-	78	78	78	75	-	-	-	-	-
**Total**	1154	2541	1448	529	1297	2676	1997	997	566	2894	568	2731	988	3233

NCS = nasal congestion score; NPS = nasal polyp score (endoscopic; 0–8 total); UPSIT = University of Pennsylvania Smell Identification Test; TNSS = Total Nasal Symptom Score; Lund = Lund–Mackay CT score; NP Surgery = need for sinonasal/polyp surgery; SCS Use = systemic corticosteroid use; ACQ-6 = Asthma Control Questionnaire-6; AQLQ = Asthma Quality of Life Questionnaire; SNOT = Sino-Nasal Outcome Test; VAS-rhinorrhea / VAS-loss of smell / VAS-nasal symptoms = visual analogue scales for the indicated domains; AEs = adverse events.

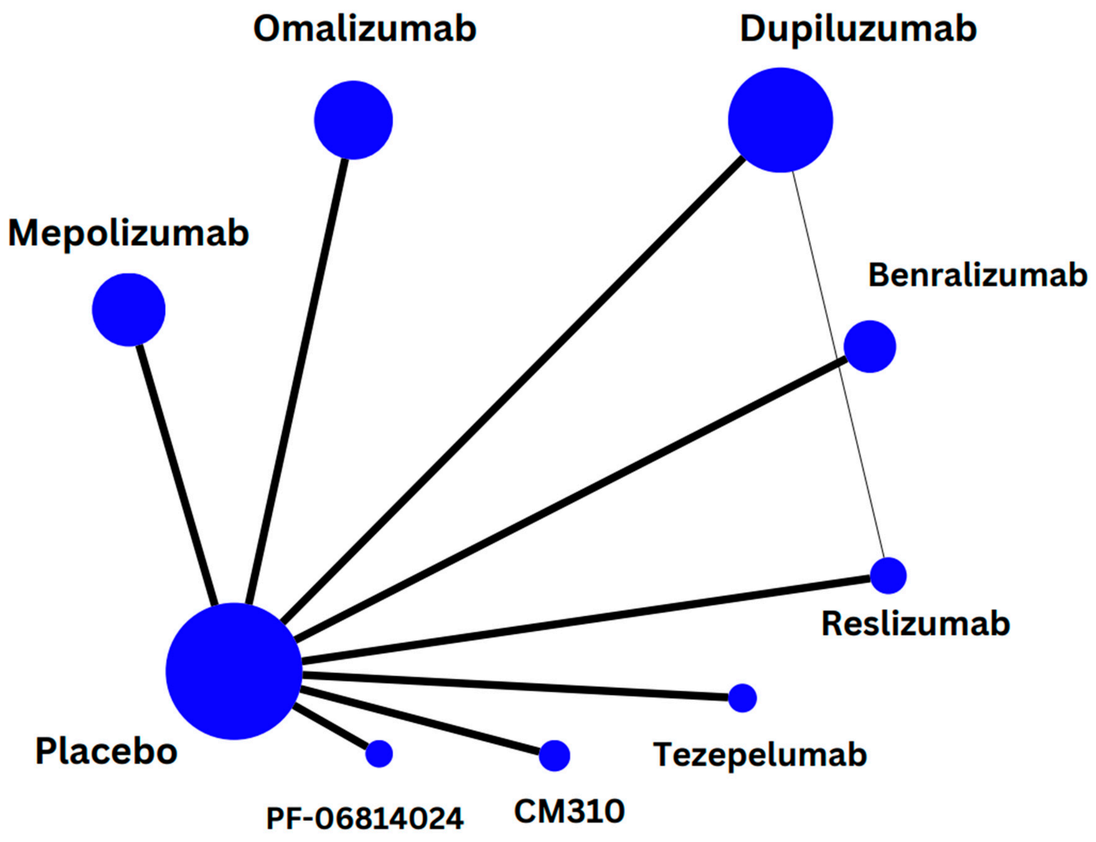

Figure S1. A graph showing the network of comparisons between biological therapies for CRSwNP in the literature
